# Establishment and validation of an artificial intelligence web application for predicting postoperative in-hospital mortality in patients with hip fracture: a national cohort study of 52 707 cases

**DOI:** 10.1097/JS9.0000000000001599

**Published:** 2024-05-15

**Authors:** Mingxing Lei, Taojin Feng, Ming Chen, Junmin Shen, Jiang Liu, Feifan Chang, Junyu Chen, Xinyu Sun, Zhi Mao, Yi Li, Pengbin Yin, Peifu Tang, Licheng Zhang

**Affiliations:** a Department of Orthopedics, National Clinical Research Center for Orthopedics, Sports Medicine and Rehabilitation, PLA General Hospital; b Department of Orthopedics, Chinese PLA General Hospital; c Chinese PLA Medical School; d Department of Emergency, The First Medical Center of PLA General Hospital, Beijing; e Department of Orthopedics, Hainan Hospital of Chinese PLA General Hospital, Hainan, People’s Republic of China

**Keywords:** artificial intelligence, hip fracture, in-hospital mortality, national cohort study, prediction model

## Abstract

**Background:**

In-hospital mortality following hip fractures is a significant concern, and accurate prediction of this outcome is crucial for appropriate clinical management. Nonetheless, there is a lack of effective prediction tools in clinical practice. By utilizing artificial intelligence (AI) and machine learning techniques, this study aims to develop a predictive model that can assist clinicians in identifying geriatric hip fracture patients at a higher risk of in-hospital mortality.

**Methods:**

A total of 52 707 geriatric hip fracture patients treated with surgery from 90 hospitals were included in this study. The primary outcome was postoperative in-hospital mortality. The patients were randomly divided into two groups, with a ratio of 7:3. The majority of patients, assigned to the training cohort, were used to develop the AI models. The remaining patients, assigned to the validation cohort, were used to validate the models. Various machine learning algorithms, including logistic regression (LR), decision tree (DT), naïve bayesian (NB), neural network (NN), eXGBoosting machine (eXGBM), and random forest (RF), were employed for model development. A comprehensive scoring system, incorporating 10 evaluation metrics, was developed to assess the prediction performance, with higher scores indicating superior predictive capability. Based on the best machine learning-based model, an AI application was developed on the Internet. In addition, a comparative testing of prediction performance between doctors and the AI application.

**Findings:**

The eXGBM model exhibited the best prediction performance, with an area under the curve (AUC) of 0.908 (95% CI: 0.881–0.932), as well as the highest accuracy (0.820), precision (0.817), specificity (0.814), and F1 score (0.822), and the lowest Brier score (0.120) and log loss (0.374). Additionally, the model showed favorable calibration, with a slope of 0.999 and an intercept of 0.028. According to the scoring system incorporating 10 evaluation metrics, the eXGBM model achieved the highest score (56), followed by the RF model (48) and NN model (41). The LR, DT, and NB models had total scores of 27, 30, and 13, respectively. The AI application has been deployed online at https://in-hospitaldeathinhipfracture-l9vhqo3l55fy8dkdvuskvu.streamlit.app/, based on the eXGBM model. The comparative testing revealed that the AI application’s predictive capabilities significantly outperformed those of the doctors in terms of AUC values (0.908 vs. 0.682, *P*<0.001).

**Conclusions:**

The eXGBM model demonstrates promising predictive performance in assessing the risk of postoperative in-hospital mortality among geriatric hip fracture patients. The developed AI model serves as a valuable tool to enhance clinical decision-making.

## Introduction

Highlights

- Artificial intelligence (AI)-based predictive model shows promising performance in assessing in-hospital mortality risk for geriatric hip fracture patients.
- eXGBM model achieves highest prediction accuracy, precision, specificity, and F1 score among various machine learning algorithms.
- The AI application outperforms doctors in predicting in-hospital mortality with significantly higher area under the curve values.
- Developed AI model serves as a valuable tool to enhance clinical decision-making in managing hip fracture patients.
- Online deployment of the AI application provides accessible and efficient prediction capabilities.

Hip fractures are a common and serious injury among the geriatric population, resulting in significant morbidity and mortality^1^. It is a significant public health issue, affecting individuals, healthcare systems, and economies globally^2^. With an aging population, the prevalence and burden of hip fractures are expected to rise^2,3^. In-hospital mortality following hip fractures is a major concern, and accurate prediction of this outcome is crucial for appropriate clinical management and resource allocation^4^.

The early prediction of in-hospital mortality in geriatric hip fracture patients has been challenging, but is of utmost importance. Because identifying patients at higher risk of mortality can help healthcare providers prioritize their care and implement preventive measures^5,6^. Traditionally, prediction models have relied on clinical risk factors and scoring systems, which have demonstrated moderate accuracy^5^. However, recent advancements in artificial intelligence (AI) and machine learning techniques offer new opportunities for improving mortality prediction in this population^5^.

Machine learning algorithms have the ability to analyze large and complex datasets, identify patterns, and generate predictive models. These algorithms can incorporate multiple variables to develop accurate and personalized predictions^7,8^. By harnessing the power of machine learning, we can potentially enhance the early identification of high-risk patients and improve clinical decision-making in the management of geriatric hip fractures^5^. The use of machine learning algorithms in the field of hip fracture prediction has shown promising results^4,5,9^. Studies have demonstrated the potential of these models in diagnosing hip fractures^4,9^ and predicting outcomes^5^. However, limited research has focused specifically on predicting in-hospital mortality.

Therefore, this study aims to develop and validate an AI model using machine learning techniques to predict the risk of postoperative in-hospital mortality in geriatric hip fracture patients. The findings of this study have the potential to significantly impact clinical practice by providing clinicians with a valuable tool to assess mortality risk and guide treatment decisions.

## Patients and methods

### Patients

A total of 52 707 geriatric patients with hip fractures treated surgically in 90 hospitals from January 2011 to September 2021 were retrospectively included in this study. Patients with a diagnosis of hip fracture based on ICD-9-CM codes (820.x) or ICD-10-CM codes (S72.x) were extracted. We collected the data through a comprehensive review of electronic medical records based on standardizing data collection procedures across all participating hospitals. The participating hospitals were located in various regions of the country, ensuring a broad representation of healthcare settings. In this study, only patients without missing data were included for analysis. Patients were excluded from the analysis if they were not surgically treated, were under the age of 60, or had an unclear fracture type in the hip. The patient flowchart is depicted in Supplementary Figure 1 (Supplemental Digital Content 1, http://links.lww.com/JS9/C559).

In this study, the ‘Military Medical and Health No. 1’ system was used to extract data, and the system’s reliability stems from its ability to gather comprehensive and accurate information from a wide range of medical facilities within the alliance hospitals. By enabling the unified extraction of data from multiple hospitals through a standardized system, we could access a larger, more diverse dataset, enhancing the generalizability and robustness of our findings. Moreover, this approach ensured data consistency and accuracy across all participating hospitals, minimizing errors and bias in the research. Data cleaning procedures and regular quality checks were performed to maintain data accuracy and completeness. A secure data management system was established, and monitoring and auditing processes were conducted to identify and address any issues.

**Figure 1:**
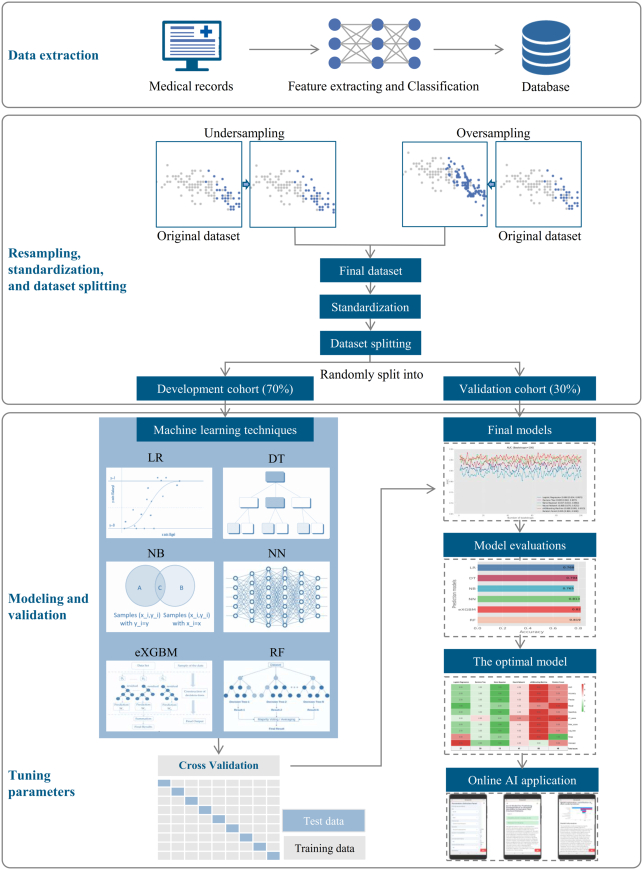
Study design and machine learning process. The experimental design consisted of three main components: data collection, resampling and randomization, and modeling and validation. The study employed six techniques for modeling. including the Logistic Regression (LR) model, as well as five machine learning models: Decision Tree (DT), Naïve Bayesian (NB), Neural Network (NN), eXGBoosting Machine (eXGBM), and Random Forest (RF)

The included patients were randomly divided into two groups, with a 7:3 ratio^10^. The training cohort, consisting of the majority of patients, was used for model development, while the validation cohort, consisting of the remaining patients, was used for validation. The study design is illustrated in Figure 1. This study was registered and approved by the Ethics Committee of our hospital, and this study also registered at a national clinical trial registry. All data were analyzed anonymously, and the study complied with the principles outlined in the Helsinki Declaration. Written informed consent was obtained from all patients, and the study adhered to the STROCSS criteria (Supplemental Digital Content 2, http://links.lww.com/JS9/C560)^11^ and the TRIPOD Checklist^12^.

### Variables and outcome

Demographic information (age and sex), fracture type, surgical procedure, and a range of comorbidities (number of comorbidities, anemia, hypertension, coronary heart disease, cerebrovascular disease, heart failure, atherosclerosis, renal failure, nephrotic syndrome, respiratory system disease, gastrointestinal bleeding, gastrointestinal ulcer, liver failure, cirrhosis, gastritis, diabetes, dementia, and cancer) were collected based on the availability of data. Diagnosis was determined by physicians at each institution, following standardized diagnostic guidelines. The primary outcome was in-hospital mortality after surgery. In the study, we collated data on various features, encompassing patient demographics, fracture types, and diverse pre-existing conditions, upon the patient’s admission. The outcome measure, in-hospital death, was subsequently recorded following the surgical intervention during the hospital stay. By examining a broad spectrum of variables, this study aims to offer a comprehensive insight into the factors that contribute to in-hospital mortality among elderly patients with hip fracture.

### Data process

The SMOTETomek resampling strategy^13,14^ was employed to address the issue of imbalanced data and generate robust models. SMOTETomek combines the Synthetic Minority Oversampling Technique with Tomek Links Undersampling to create a new dataset with a larger sample size and a more balanced distribution. This strategy enhances the statistical power and generalizability of the findings, providing a solid foundation for further analyses and model development. Additionally, a data preprocessing pipeline was utilized to ensure consistent and reproducible data transformation, thereby improving the accuracy and reliability of the machine learning models. The scikit-learn library was used for data preprocessing pipelines. A stratified strategy was implemented to maintain consistent outcome class proportions in the sub-datasets.

### Modeling

Various machine learning algorithms, including logistic regression (LR), decision tree (DT), naïve bayesian (NB), neural network (NN), eXGBoosting machine (eXGBM), and random forest (RF), were used to model development. Each model received the same input features to ensure consistency. Grid and random hyperparameter searches, combined with fivefold cross-validation, were performed to identify the optimal hyper-parameters for each model. The area under the curve (AUC) was used as the optimization metric. To account for variability in model performance, a broad range of upper and lower bounds for the hyper-parameters was set in the search, resulting in a mix of underfitted and overfitted models.

### Validation

The models were validated using a variety of evaluation metrics, including AUC, accuracy, precision, specificity, recall, F1 score, Brier score, log loss, calibration slope, and intercept. AUC was calculated after applying 100 bootstraps. Calibration curve, density curve, and decision curve were utilized to evaluate the calibration ability, discriminative ability, and clinical net benefits of the models, respectively. Furthermore, a scoring system was developed based on previous studies to comprehensively evaluate the predictive performance of the models. The scoring system incorporated 10 evaluation metrics, with higher scores indicating superior predictive performance (range: 0–60).

Confusion matrix was used to determine the accuracy, precision, recall (sensitivity), and specificity of the models.


Accuracy=(TP+TN)/(TP+FN+FP+TN)

Precision=TP/(TP+FP)

Specificity=1−FP/(TN+FP)

Recall(sensitivity)=TP/(TP+FN)

The confusion matrix is typically presented as a table with four main components: true positives (TP), true negatives (TN), false positives (FP), and false negatives (FN). Each component represents a specific outcome of the model’s predictions.

The Brier score, as an overall performance measure, was calculated using the following equation. It is calculated by summing the squared differences between the predicted probability and the actual probability for each sample, divided by the total sample size.


$$\mathrm{Brier Score}=\;\frac{1}{N}\sum_{i=1}^{n}{\left(p_{i}-o_{i}\right)}^{2}$$

where, N represents the total sample, $p_{i}$ represents the predicted risk of postoperative in-hospital mortality, and $o_{i}$ represents the actual probability of postoperative in-hospital mortality.

The log loss formula, commonly used in classification model evaluation, measures the quality of predictions made by the model. It calculates the negative logarithm of the predicted probability for each class, multiplied by the true label. The sum of these values is then divided by the total number of samples.


$$\mathrm{Log Loss}=-\;\frac{1}{N}\sum_{i=1}^{N}\sum_{j=1}^{M}y_{\mathrm{ij}}\log(p_{\mathrm{ij}})$$

where, N represents the number of samples, M represents the number of classes, $y_{\mathrm{ij}}$ represents the true label of sample i for class j (0 or 1), and $p_{\mathrm{ij}}$ represents the predicted probability of sample i belonging to class j .

We extracted data from a medical center in the United State (Medical Information Mart for Intensive Care III [MIMIC-III] database)^15^, and the data on patients with hip fracture were collected as an external validation cohort. Based on the same inclusive and exclusive criteria in the study, a series of 246 patients were included for analysis. Patient’s flowchart baseline characteristics of patients, and detailed information are summarized in Supplementary File 1 (Supplemental Digital Content 3, http://links.lww.com/JS9/C561). The utilization of the MIMIC-III database was authorized by the institutional review board of a medical center in another nation. As the data within the database has been de-identified, patient consent was not necessary. This study pledged to scrupulously adhere to ethical guidelines and legal regulations throughout the research process, assuring the appropriate use and robust protection of patient privacy.

### Feature importance

Feature importance was employed to enhance the interpretability of machine learning models^16,17^, as it allows for the quantification of the relative importance of each feature in the model’s predictions. SHAP is a unified framework designed to interpret machine learning predictions, and it serves as a novel approach to explain various black-box machine learning models^18^. By identifying the most influential features, clinicians can better understand the factors that contribute the most to the outcome, making the model’s predictions more interpretable and easier to validate. In the present study, Shapley additive explanation (SHAP) values were employed to determine the importance of each input parameter. SHAP values quantify the contribution of each feature to the model’s output.


$$g\left(z^{\prime }\right)=\phi_{0}+\sum_{j=1}^{M}\phi_{j}{Z^{\prime }}_{j}$$

In the context of this explanation, certain variables were defined to describe the interpretation model. The variable g symbolizes the interpretation model itself, representing its underlying principles and mechanisms. M denotes the total number of input parameters in the model, $\phi_{0}$ signifies a constant value within the model. Furthermore, $\phi_{j}$ embodies the Shapley value assigned to each specific feature of the model, capturing its contribution to the overall interpretation, and ${Z^{\prime }}_{j}$ represents the coalition vector.

Among the coalition vectors, their values play a critical role in providing insight into the interpretability of the model. A value of ‘1’ denotes that a particular feature in the coalition vector aligns with the corresponding feature of the case x being explained. Conversely, a value of ‘0’ indicates that the feature is absent in the present case x . By applying this concept to our analysis, we can consider case x as a scenario where all simplified features hold a value of 1. With this assumption, the SHAP expression can be further simplified and outlined as follows:


$$g\left(x^{\prime }\right)=\phi_{0}+\sum_{j=1}^{M}\phi_{j}$$

### Individual prediction

In the present study, we employed the SHAP method to perform individual prediction, aiming to offer a comprehensive insight into the prediction-making process of the model at an individual level^10,19^. By utilizing the trained model, we calculated SHAP values for each feature within the dataset. These SHAP values signify the average marginal contribution of a feature towards the model’s predictions. They span from −1 to 1, with −1 denoting a negative contribution and 1 indicating a positive contribution. To render the SHAP values comprehensible, we employed a waterfall plot. This visual aid aided in identifying the crucial features for the model’s predictions and comprehending their individualized contributions towards the predictions.

### Development of an online AI application

The online AI application was developed using the powerful combination of *Streamlit* and *GitHub*. This interactive AI application encompasses various elements that enhance its usability and functionality. One notable component was a user-friendly panel that allowed individuals to select model parameters from a comprehensive list of seventeen significant variables. This empowered users to personalize their inputs and tailor them to their specific scenario. The application also included a dedicated interface for calculating the probability of postoperative in-hospital mortality based on the chosen parameters. By leveraging this interface, users could gain valuable insights and obtain an anticipated probability for this outcome.

To promote a thorough understanding, an additional panel was incorporated that showcases the contribution of each model predictor towards the ultimate outcome, employing the SHAP method. This component sheds light on the significance and influence of various features within the model. By leveraging the AI platform and examining the contribution of model features to predictions for each individual case, researchers can pinpoint variables that act as protective or risk factors for specific patients. Moreover, the AI platform assigns priority rankings to variables on an individualized basis, emphasizing the crucial risk factors that necessitate heightened clinical attention. Moreover, the application prioritizes clarity by providing an interface solely dedicated to introducing and explaining the model. This section offers in-depth information about the model’s methodology, principles, and underlying factors. It serves as a valuable resource for users seeking a detailed understanding of how the model operates. Furthermore, the application classifies patients into high-risk or low-risk groups based on the optimal risk threshold. This categorization enhances the utility of the application by providing actionable insights that can guide medical decision-making.

### Comparative evaluation of prediction performance: humans vs. the AI application

To thoroughly assess and compare prediction performance, we embarked on a comprehensive study pitting doctors against an AI application. Eight highly esteemed doctors, known for their expertise, willingly took part in this study. Each doctor autonomously provided predictions on the risk of death during hospitalization, drawing from their own assessments. Through this comparative analysis, our objective was to gain valuable insights into the relative performance and effectiveness of human experts versus the AI platform. In order to ensure a fair evaluation, we established a standardized set of criteria for both the doctors and the AI platform. These criteria encompassed factors such as AUC, accuracy, precision, recall, and overall predictive power. By employing these metrics, we aimed to gage the true capabilities of both human doctors and the AI application in accurately predicting the risk of death. The study was conducted in a controlled environment, with each doctor and the AI platform being presented with the same dataset of patient records. The doctors meticulously reviewed the medical histories, conducted thorough examinations, and utilized their extensive knowledge and experience to make their predictions. On the other hand, the AI platform employed advanced algorithms and machine learning techniques to analyze the data and generate predictions.

### Statistical analysis

Categorical variables were presented as proportions and compared using the *χ* ^2^ test in subgroup analysis. Python (version 3.9.7) was used for machine learning algorithms and hyperparameter tuning based on scikit-learn (version 1.2.2). R language program (version 4.1.2) was employed for all statistical analyses. A significance level of less than 0.05 was considered statistically significant.

## Results

### Patient’s demographics

Table 1 provides an overview of the baseline characteristics of the patients included in the study. The total number of patients in the study was 52 707. In terms of age distribution, 25.2% of the patients were in the 60–69 age range, while 43.8% were aged 70–79. Patients aged 80–89 accounted for 27.5%, and 3.3% were in the 90–100 age range. A small portion of patients (0.1%) were aged 100 or above. The sex distribution showed that 36.4% of the patients were male and 63.6% were female. Fracture type analysis revealed that 57.4% of the patients had femoral neck fractures, while 42.6% had intertrochanteric fractures. In terms of the type of operation performed, hip joint replacement accounted for 51.7%, while internal fixation made up 48.3%. The number of comorbidities varied among the patients. Approximately half (49.1%) had no comorbidities. The percentages of patients with 1, 2, and three or more comorbidities were 23.3, 22.6, and 4.9%, respectively.

**Table 1:** A comparison of clinical characteristics between patients with and without in-hospital death.

		In-hospital death		
Characteristics	Overall	No	Yes	*P*
*n*	52 707	52 257	450	
Age (%)				<0.001
60–69	13 285 (25.2)	13 241 (25.3)	44 (9.8)	
70–79	23 093 (43.8)	22 932 (43.9)	161 (35.8)	
80–89	14 520 (27.5)	14 325 (27.4)	195 (43.3)	
90–100	1753 (3.3)	1703 (3.3)	50 (11.1)	
>100	56 (0.1)	56 (0.1)	0 (0.0)	
Sex (male/female, %)	19 200/33 507 (36.4/63.6)	18 969/33 288 (36.3/63.7)	231/219 (51.3/48.7)	<0.001
Fracture type (femoral neck fracture/intertrochanteric fracture, %)	30 251/22 456 (57.4/42.6)	30 018/22 239 (57.4/42.6)	233/217 (51.8/48.2)	0.018
Operation (Hip joint replacement/Internal fixation, %)	27 274/25 433 (51.7/48.3)	27 019/25 238 (51.7/48.3)	255/195 (56.7/43.3)	0.040
Number of comorbidities (%)				<0.001
0	25 904 (49.1)	25 886 (49.5)	18 (4.0)	
1	12 299 (23.3)	12 220 (23.4)	79 (17.6)	
2	11 933 (22.6)	11 742 (22.5)	191 (42.4)	
≧3	2571 (4.9)	2409 (4.6)	162 (36.0)	
Anemia (no/yes, %)	52 362/345 (99.3/0.7)	51 916/341 (99.3/0.7)	446/4 (99.1/0.9)	0.745
Hypertension (no/yes, %)	47 865/4842 (90.8/9.2)	47 467/4790 (90.8/9.2)	398/52 (88.4/11.6)	0.096
Coronary disease (no/yes, %)	50 865/1842 (96.5/3.5)	50 481/1776 (96.6/3.4)	384/66 (85.3/14.7)	<0.001
Cerebrovascular disease (no/yes, %)	50 987/1720 (96.7/3.3)	50 568/1689 (96.8/3.2)	419/31 (93.1/6.9)	<0.001
Heart failure (no/yes, %)	52 541/166 (99.7/0.3)	52 108/149 (99.7/0.3)	433/17 (96.2/3.8)	<0.001
Atherosclerosis (no/yes, %)	52 410/297 (99.4/0.6)	51 963/294 (99.4/0.6)	447/3 (99.3/0.7)	0.999
Renal failure (no/yes, %)	52 514/193 (99.6/0.4)	52 087/170 (99.7/0.3)	427/23 (94.9/5.1)	<0.001
Nephrotic syndrome (no/yes, %)	52 701/6 (100.0/0.0)	52 252/5 (100.0/0.0)	449/1 (99.8/0.2)	0.046
Respiratory system disease (no/yes, %)	49 216/3491 (93.4/6.6)	48 932/3325 (93.6/6.4)	284/166 (63.1/36.9)	<0.001
Gastrointestinal bleeding (no/yes, %)	52 651/56 (99.9/0.1)	52 213/44 (99.9/0.1)	438/12 (97.3/2.7)	<0.001
Gastrointestinal ulcer (no/yes, %)	52 637/70 (99.9/0.1)	52 190/67 (99.9/0.1)	447/3 (99.3/0.7)	0.013
Liver failure (no/yes, %)	52 703/4 (100.0/0.0)	52 254/3 (100.0/0.0)	449/1 (99.8/0.2)	0.011
Cirrhosis (no/yes, %)	52 592/115 (99.8/0.2)	52 147/110 (99.8/0.2)	445/5 (98.9/1.1)	<0.001
Gastritis (no/yes, %)	52 652/55 (99.9/0.1)	52 204/53 (99.9/0.1)	448/2 (99.6/0.4)	0.131
Diabetes (no/yes, %)	49 613/3094 (94.1/5.9)	49 206/3051 (94.2/5.8)	407/43 (90.4/9.6)	0.001
Dementia (no/yes, %)	52 515/192 (99.6/0.4)	52 069/188 (99.6/0.4)	446/4 (99.1/0.9)	0.144
Cancer (no/yes, %)	52 117/590 (98.9/1.1)	51 679/578 (98.9/1.1)	438/12 (97.3/2.7)	0.004

### A comparison of clinical characteristics stratified by postoperative in-hospital death

The study further compared the clinical characteristics between patients who experienced in-hospital death and those who did not (Table 1). Age, sex, fracture type, operation type, and the number of comorbidities showed significant differences between the two groups (*P*<0.001, *P*<0.001, *P*=0.018, *P*=0.040, and *P*<0.001, respectively). In detail, older age, male, patients with intertrochanteric fractures, those who underwent hip joint replacement, and a higher number of comorbidities had slightly higher in-hospital mortality rates. In addition, patients with variables comorbidities, including coronary heart disease (*P*<0.001), cerebrovascular disease (*P*<0.001), heart failure (*P*<0.001), renal failure (*P*<0.001), nephrotic syndrome (*P*=0.046), respiratory system disease (*P*<0.001), gastrointestinal bleeding (*P*<0.001), gastrointestinal ulcer (*P*=0.013), liver failure (*P*=0.011), cirrhosis (*P*<0.001), diabetes (*P*=0.001), and cancer (*P*=0.004), were more likely to experience in-hospital mortality than patients without these comorbidities after surgery. Thus, the above 17 variables were used as predictors in the model. Due to the significant predictive value of these factors in determining mortality after hip fracture^20–26^, they were utilized in the development of a prediction model. Subgroup analysis was also conducted based on age (Supplementary Table 1, Supplemental Digital Content 4, http://links.lww.com/JS9/C562), sex (Supplementary Table 2, Supplemental Digital Content 5, http://links.lww.com/JS9/C563), and the number of comorbidities (Supplementary Table 3, Supplemental Digital Content 6, http://links.lww.com/JS9/C564).

### Model evaluation

**Figure 2:**
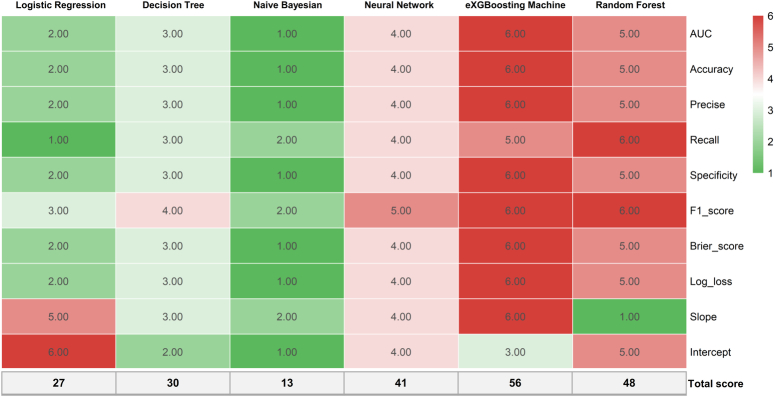
Heatmap of the scoring system for comprehensively evaluating the prediction performance of all models. The scoring system incorporated 10 evaluation metrics, and each metric was rated on a scale of 1 to 6, where higher scores denoted superior predictive performance. In this visualization, green represents relatively poor prediction performance, while red represents relatively good prediction performance.

**Figure 3:**
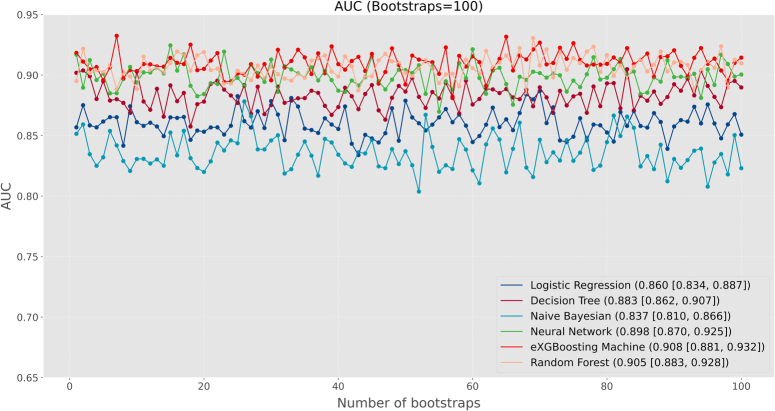
Area under the curve (AUC) achieved by different models after applying 100 bootstraps. The *X*-axis of the graph represents the number of bootstraps, while the *Y*-axis represents the AUC value. Each model is represented by a different color.

**Figure 4:**
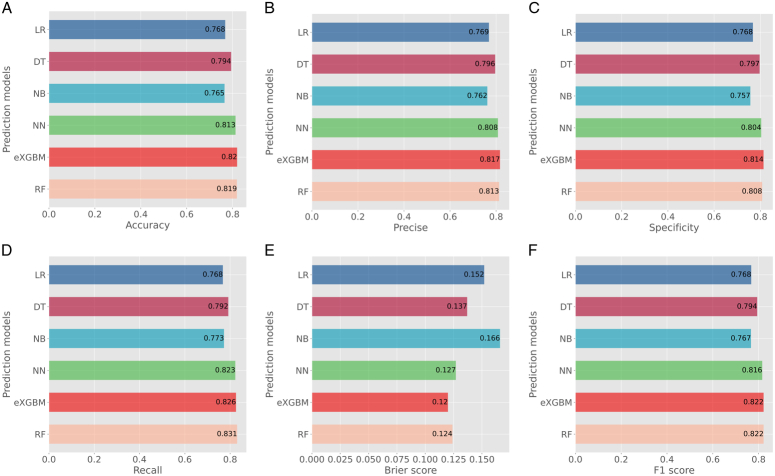
Prediction metrics for all the models. A. Accuracy; B. Precise; C. Specificity; D. Recall; E. Brier score; F. F1 score. Each model is represented by a different color.

**Figure 5:**
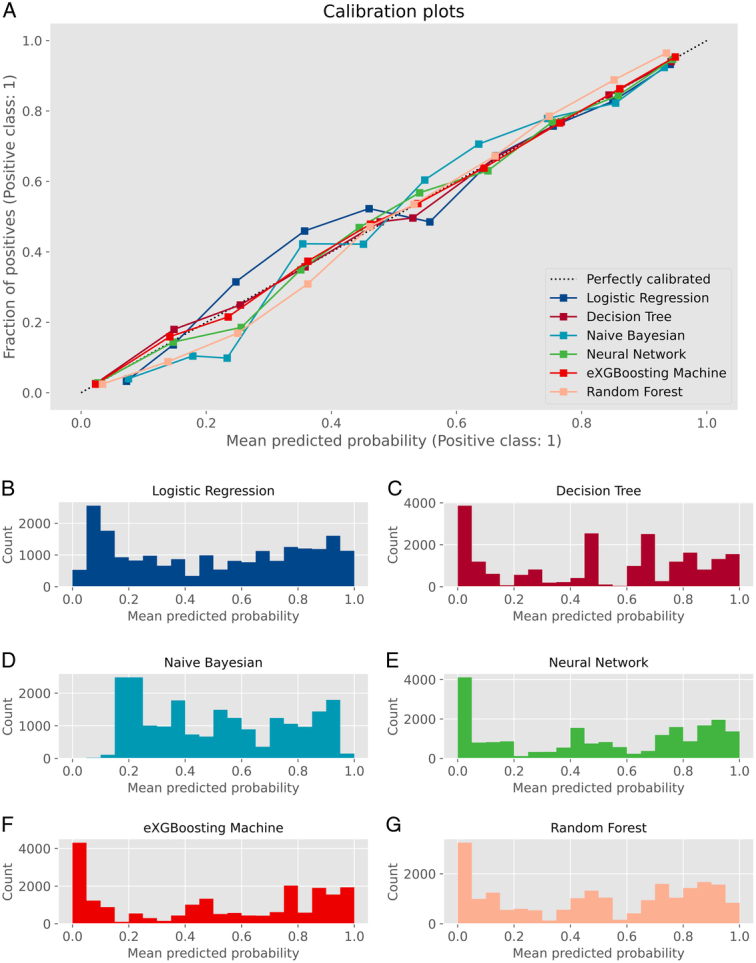
Calibration curves and histograms of mean prediction probabilities for all the models. A. Calibration curve; B. Logistic Regression; C. Decision Tree; D. Naïve Bayesian; E. Neural Network; F. eXGBoosting Machine; G. Random Forest. The calibration curve plots the mean predicted probability against the fraction of positives in deciles. This curve provides insights into the calibration or alignment of the predicted probabilities with the actual probabilities. Additionally, the histogram displays the mean predicted probability along with its corresponding count.

**Figure 6:**
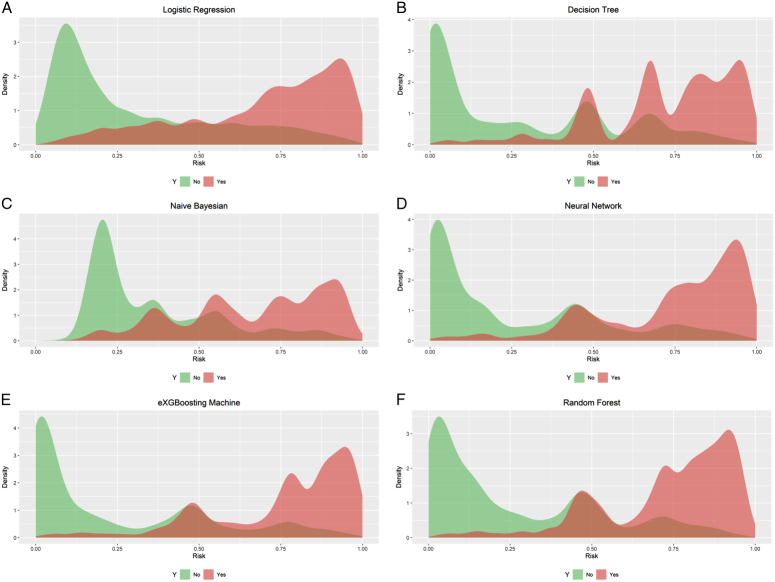
Density curves for all the models. A. Logistic Regression; B. Decision Tree; C. Naïve Bayesian; D. Neural Network; E. eXGBoosting Machine; F. Random Forest. The green indicates patients without in-hospital mortality, and the red indicates patients with in-hospital mortality. The less overlap between the red and green colors, the better the model’s ability to discriminate.

**Figure 7:**
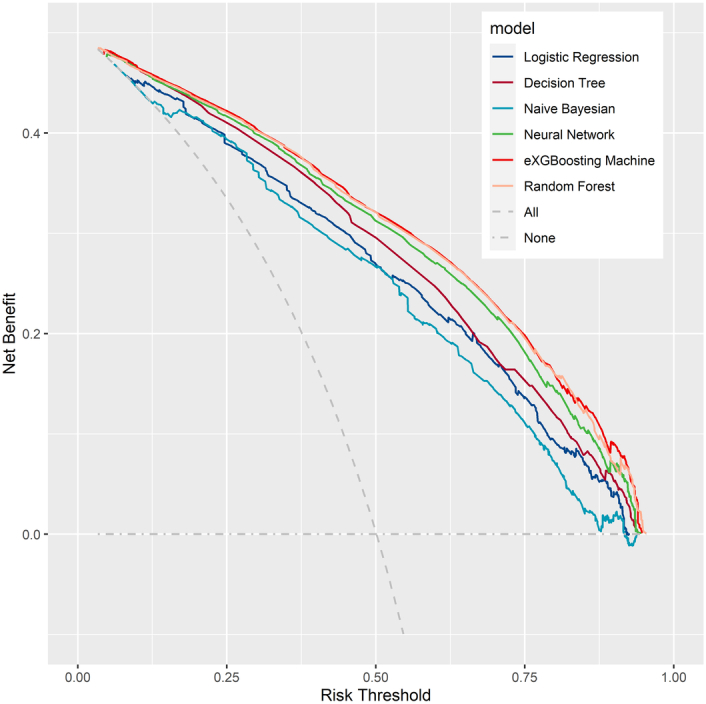
Decision curve analysis for all the models in the validation cohort. The horizontal dotted gray line represents the ‘treat none’ scenario, indicating the net benefit when no treatment is given. On the other hand, the dotted inclined gray line represents the ‘treat all’ scenario, indicating the net benefit of different threshold probability when all individuals receive treatment.

Based on the scoring system, the eXGBM model demonstrated the highest total score of 56 (Fig. 2), indicating superior predictive performance. It was followed by the RF model with a total score of 48, and the NN model with a total score of 41. The LR, DT, and NB models achieved total scores of 27, 30, and 13, respectively. Notably, among the six models, the eXGBM model exhibited the highest AUC [0.908 (95% CI: 0.881–0.932), Fig. 3], while the RF model obtained the second highest AUC [0.905 (95% CI: 0.883–0.928)]. In contrast, the AUC values for the LR and NB models were 0.860 (95% CI: 0.834–0.887) and 0.837 (95% CI: 0.810–0.866), respectively (Fig. 3 and Table 2). Additionally, the eXGBM model outperformed the other models in terms of accuracy (0.820), precision (0.817), specificity (0.814), F1 score (0.822), Brier score (0.120), and log loss (0.374) (Fig. 4). Calibration curve analysis further demonstrated the favorable calibration ability of the eXGBM model (Fig. 5), with a slope of 0.999 and an intercept of 0.028 (Supplementary Figure 2, Supplemental Digital Content 7, http://links.lww.com/JS9/C565). Density curves were used to assess the predictive ability of distinguishing the postoperative in-hospital mortality risk between two groups (Fig. 6). The eXGBM, RF, and NN models displayed relatively small overlapping regions and larger distinguishment areas, with the green peak located on the left side of the *x*-axis and the red peak located on the right side of the *x*-axis. However, the LR, DT, and NB models exhibited relatively larger areas of overlap. Decision curves (Fig. 7 and Supplementary Figure 3, Supplemental Digital Content 8, http://links.lww.com/JS9/C566) indicated that the eXGBM and RF models offered relatively better clinical net benefits compared to other machine learning models. These findings support the designation of the eXGBM model as the optimal choice for assessing the risk of postoperative in-hospital death among patients with geriatric hip fracture. The AUC value of the model was 0.784 (95% CI: 0.699–0.869) in the external validation cohort, indicating relatively favorable prediction even in the ICU settings. The accuracy was 0.907, Brier score was 0.095, and Log loss was 0.464. More detailed information was summarized in Supplementary File 1 (Supplemental Digital Content 3, http://links.lww.com/JS9/C561). The model in our study was established based on patients in the general ward, but it still could achieve a favorable AUC value in the ICU population. This indicated that the model also had a good extrapolation effect and could predict outcomes effectively across different patient populations, showcasing its robustness and clinical relevance.

**Table 2:** Prediction performance of machine learning-based and traditional models in the validation set.

Models	AUC (95%CI)	Accuracy	Precise	Recall	Specificity	F1 score	Brier score	Log loss	Calibration slope	Intercept-in-large
LR	0.860 [0.834, 0.887]	0.768	0.769	0.768	0.768	0.768	0.152	0.466	0.995	0.019
DT	0.883 [0.862, 0.907]	0.794	0.796	0.792	0.797	0.794	0.137	0.424	0.981	0.030
NB	0.837 [0.810, 0.866]	0.765	0.762	0.773	0.757	0.767	0.166	0.508	1.173	-0.073
NN	0.898 [0.870, 0.925]	0.813	0.808	0.823	0.804	0.816	0.127	0.397	0.992	0.026
eXGBM	0.908 [0.881, 0.932]	0.820	0.817	0.826	0.814	0.822	0.120	0.374	0.999	0.028
RF	0.905 [0.883, 0.928]	0.819	0.813	0.831	0.808	0.822	0.124	0.389	1.212	-0.023

AUC, Area Under the Curve; CI, Confident Interval; LR, Logistic Regression; DT, Decision Tree; NB, Naïve Bayesian; eXGBM, eXGBoosting Machine; RF, Random Forest.

**Table 3:** Prediction of mortality among hip fracture patients using machine learning according to current literature.

Authors	Published years	Study design	Number of patients	Age of patients	Interventions	Machine learning techniques	Best model	Outcome (AUC)
Kitcharanant *et al.* ^24^	2022	Retrospective	492	Mean (SD): 81.55 (10.10) years	Surgery or conservative treatment (6.5%)	Gradient Boosting, Random Forests, Artificial Neural Network, Logistic Regression, Naive Bayes, Support Vector Machine, and K-Nearest Neighbors	Random Forest	1-year mortality: 0.99
Forssten *et al.* ^25^	2021	Retrospective	124 707	50 years or above	Surgery	Support Vector Machine, Naïve Bayes, Random Forest, and Logistic Regression	Logistic Regression	1-year mortality: 0.74
Oosterhoff *et al.* ^28^	2022	Retrospective	2478	65 years or above	Surgery	Stochastic Gradient Boosting, Random Forest, Support Vector Machine, Neural Network, and Penalized Logistic Regression	Stochastic Gradient Boosting and Penalized Logistic Regression	90-day mortality: 0.74; 2-year mortality: 0.70
Dijkstra *et al.* ^29^	2023	Prospective	2388	50 years or above	Surgery	Bayes Point Machine, Boosted Decision Tree, Jungle Decision Algorithm, Penalized Logistic Regression, Neural Network, and Support Vector Machine	Penalized Logistic Regression	90-day mortality: 0.80; 1-year mortality: 0.76
Zhang *et al.* ^32^	2019	Retrospective	448	60 years or above	Surgery	Bayesian belief network	Bayesian belief network	1-year mortality: 0.85
Huang *et al.* ^30^	2022	Retrospective	391	18 years or above	Not clear (Critically ill patients)	Random Forest, Gradient Boosting Machine, Decision Tree, and eXGBoosting Machine	eXGBoosting machine	In-hospital mortality: 0.797
DeBaun *et al.* ^33^	2021	Retrospective	19 835	Mean (SD): 80.6 (11.2) years	Surgery	Artificial Neural Network, Naive Bayes, and Logistic Regression	Artificial Neural Network	30-day mortality: 0.92
Li *et al.* ^34^	2021	Retrospective	1330	50 years or above	Surgery	Random Survival Forest	Random Survival Forest	30-day mortality: 0.83; 1-year mortality: 0.75
Cary *et al.* ^35^	2020	Retrospective	17 140	More than 65 years	Not clear	Logistic Regression and Multilayer Perceptron	Logistic Regression and Multilayer Perceptron	30-day mortality: 0.76; 1-year mortality: 0.75
Hjelholt *et al.* ^31^	2022	Not clear	28 791	More than 65 years	Surgery	Decision Tree	Decision Tree	1-year mortality: 0.74
Our study	2023	Retrospective	52 707	60 years or above	Surgery	Logistic Regression, Decision Tree, Naïve Bayesian, Neural Network, eXGBoosting Machine, and Random Forest	eXGBoosting Machine	In-hospital mortality: 0.908

AUC, area under the curve.

### Subgroup analysis of prediction performance

**Figure 8:**
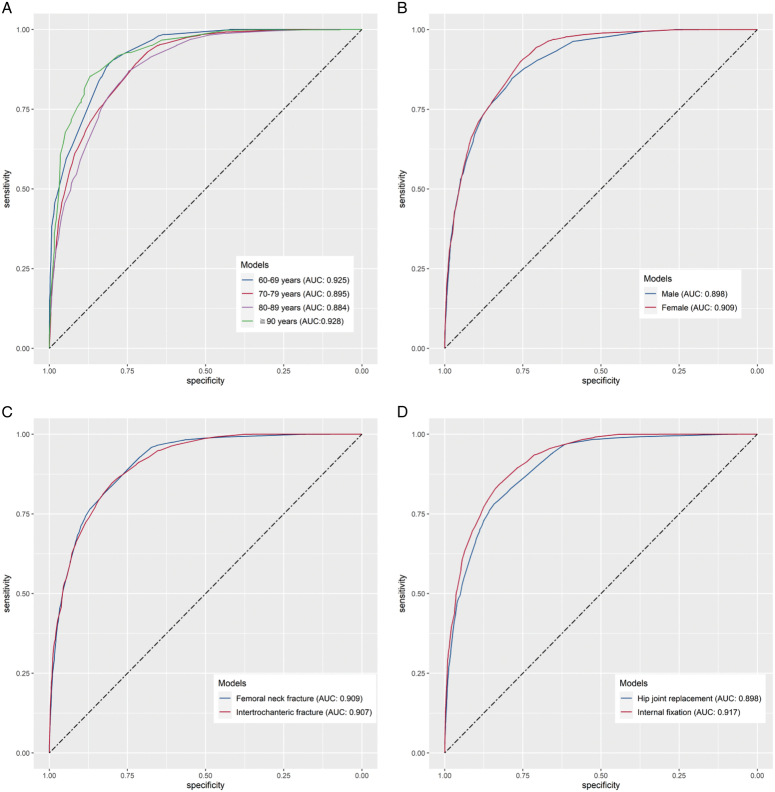
Subgroup analysis of prediction performance of the developed model. A. Age; B. Sex; C. Fracture type; D. Operation process.

We further evaluated the predictive performance of the eXGBM model in different subpopulations. The AUC value of the developed model was 0.925 (95% CI: 0.917–0.933) in patients aged 60–69, 0.895 (95% CI: 0.889–0.902) in patients aged 70–79, 0.884 (95% CI: 0.876–0.892) in patients aged 80–89, and 0.928 (95% CI: 0.911–0.946) in patients aged 90 and above (Fig. 8A). Male patients had an AUC value of 0.898 (95% CI: 0.892–0.905), while female patients had an AUC value of 0.909 (95% CI: 0.904–0.914) (Fig. 8B). Patients with femoral neck fractures had an AUC value of 0.909 (95% CI: 0.904–0.914), and patients with hip fractures had an AUC value of 0.907 (95% CI: 0.901–0.913) (Fig. 8C). Patients treated with hip joint replacement had an AUC value of 0.898 (95% CI: 0.893–0.904), and patients treated with internal fixation had an AUC value of 0.917 (0.912–0.923) (Fig. 8D). Threshold, specificity, sensitivity, accuracy, and precision are also summarized in Supplementary Table 4 (Supplemental Digital Content 9, http://links.lww.com/JS9/C567). The above results indicate that the AUC values of the model were relatively stable across different subgroups, suggesting that the model performed well in predicting outcomes in diverse population subgroups.

### Feature importance and individual prediction

Feature importance analysis using SHAP in the eXGBM model revealed that the number of comorbidities, age, and operation type were the most influential features in predicting postoperative in-hospital mortality (Supplementary Figure 4, Supplemental Digital Content 10, http://links.lww.com/JS9/C568). The result suggested that patients with a higher number of comorbidities, older age, and specific operation types were at a higher risk of in-hospital mortality following surgery.

Furthermore, we employed the SHAP method to gain detailed insights into the model’s interpretation at an individual level. By quantifying the importance of individual features in the model’s prediction, a deeper understanding of the factors influencing the outcome can be achieved. To further enhance the interpretability of the model, we presented two distinct cases. Supplementary Figure 5 (Supplemental Digital Content 11, http://links.lww.com/JS9/C569) depicts a true negative case, whereas Supplementary Figure 6 (Supplemental Digital Content 12, http://links.lww.com/JS9/C570) illustrates a true positive case. The arrows demonstrate the impact of each factor on the prediction. Blue and red arrows denote whether the factor reduces (blue) or increases (red) the risk of in-hospital death. The combined influence of all factors generates the final SHAP value, which corresponds to the prediction score. For the representative case one, the SHAP value was low (−1.90); for the representative case two, the SHAP value was high (5.07).

### Online AI prediction

**Figure 9:**
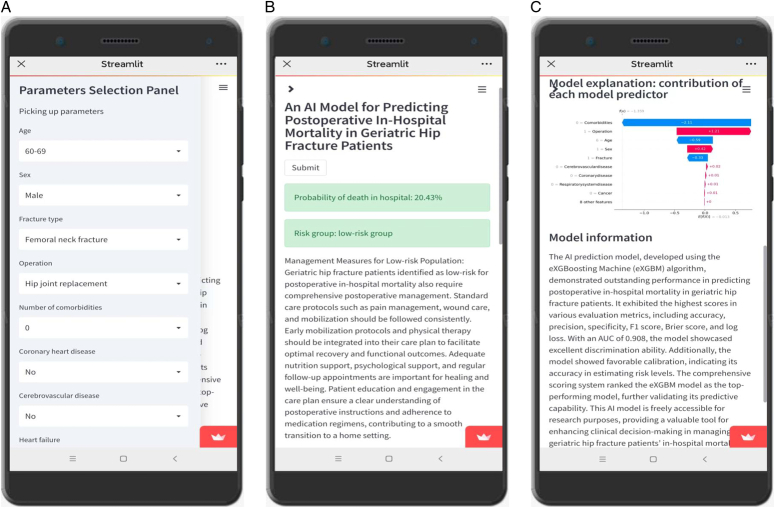
The online AI application is equipped with the optimal machine learning model. A. A panel for selecting model parameters; B. An interface for calculating the probability; B. An interface for model explanation and introduction of the model. To utilize the application, users can select the desired model parameters through the parameter selection panel. Once the parameters are set, they can simply click the ‘Submit’ button to initiate the calculation process. The application will subsequently generate and display the estimated probability of experiencing in-hospital mortality based on the chosen parameters, along with recommended therapeutic strategies.

The eXGBM model has been deployed online and was freely accessible at https://in-hospitaldeathinhipfracture-l9vhqo3l55fy8dkdvuskvu.streamlit.app/. By clicking the provided link, users can access the online AI application (Fig. 9). Once the model parameters were chosen and submitted, the risk of postoperative in-hospital mortality would be showcased. Additionally, recommendations for mitigating postoperative in-hospital mortality would be offered based on this risk assessment. Furthermore, a risk report highlighting risk or protective factors and feature importance was provided within the AI application. For instance, in the depicted case (Fig. 9), the number of comorbidities and age served as significant protective factors, while the surgical process and sex were important risk factors. In the event that the online application becomes unresponsive or inaccessible, users can reactivate it by clicking on ‘Yes, get this app back up!’. The web-based application will be operational again within ~30 s after restarting the application, allowing users to resume utilizing the application without waiting for technical support or assistance. This simple and effective approach ensures uninterrupted access to the online application.

### Comparative evaluation of prediction performance between doctors and the AI application

**Figure 10:**
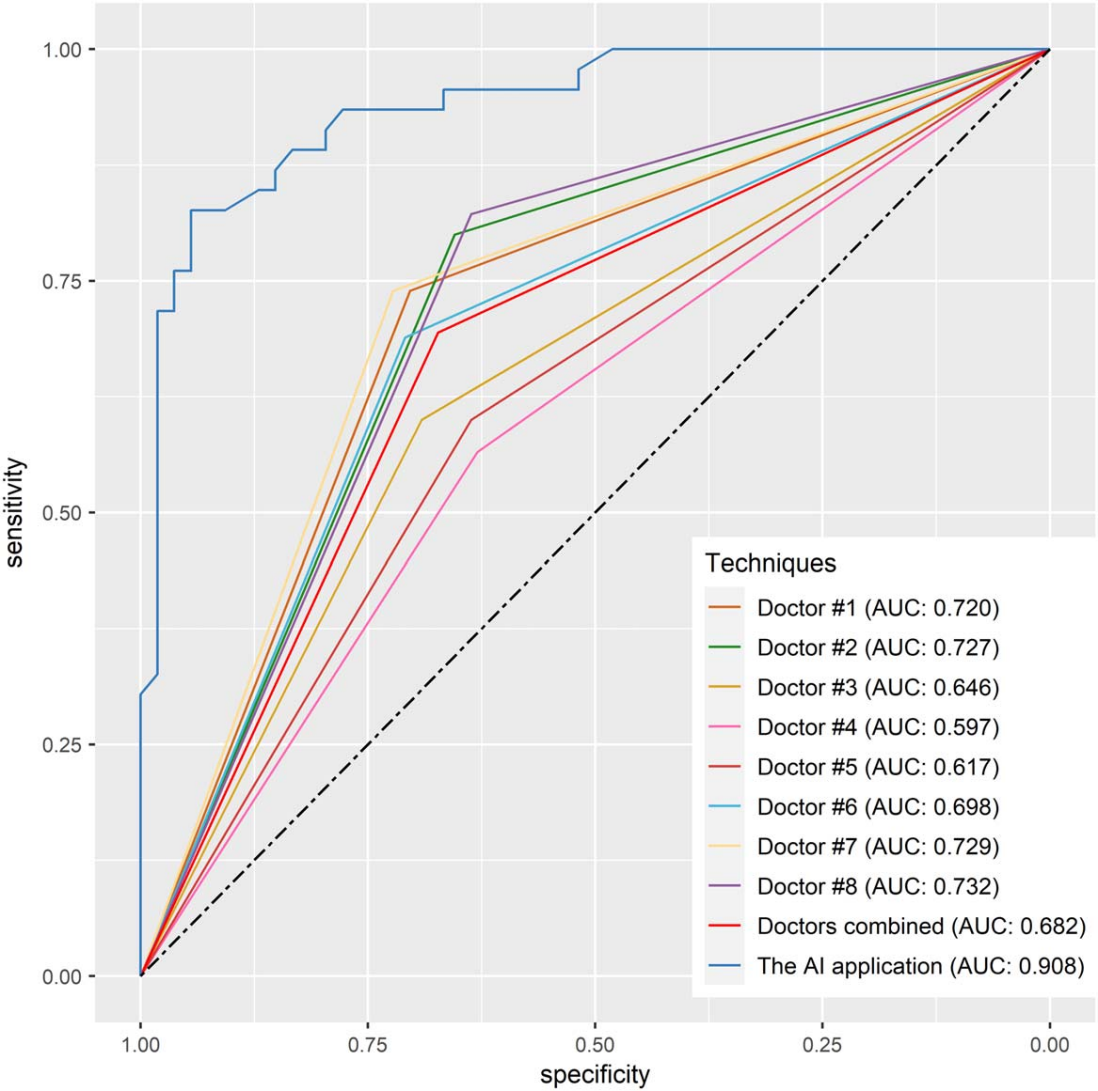
Comparative evaluation of prediction performance between doctors and the AI platform using the area under the curve (AUC) analysis.

The findings revealed intriguing insights into the comparative performance of human doctors and the AI platform. While the doctors demonstrated their expertise and ability to consider various factors beyond the dataset, the AI platform showcased its remarkable computational power and ability to process vast amounts of data quickly. In terms of accuracy, the AI platform exhibited a higher level of precision in predicting the risk of death during hospitalization with an AUC value of 0.908, while the average AUC value was 0.682 for doctors (*P*<0.001) (Fig. 10 and Supplementary Table 5, Supplemental Digital Content 13, http://links.lww.com/JS9/C571). Its ability to analyze large datasets and identify subtle patterns allowed it to make predictions with a higher degree of accuracy compared to human doctors. It is important to note that the doctors’ predictions were far behind. Furthermore, the AI platform demonstrated consistent performance across different subsets of the dataset, indicating its robustness and reliability. On the other hand, the doctors’ predictions showed some variability, highlighting the potential for subjective biases and individual differences in their assessments.

## Discussion

### Main findings

The study developed and validated an AI model using machine learning techniques to predict the risk of postoperative in-hospital mortality in geriatric hip fracture patients. Among the six models tested, the eXGBM model demonstrated the highest predictive performance, with an AUC of 0.908 and the highest scores in accuracy, precision, specificity, and F1 score. In addition, the eXGBM model achieved the highest total score according to the comprehensive scoring system, indicating superior predictive capability. The developed AI model can serve as a valuable tool to enhance clinical decision-making in assessing the risk of postoperative in-hospital mortality in geriatric hip fracture patients.

### Risk factors for predicting in-hospital mortality among hip fracture

Our study demonstrated that older age, male, patients with intertrochanteric fractures, those who underwent hip joint replacement, and comorbidities were associated with in-hospital mortality. The findings were consistent with previous studies. Previous studies have also proposed that older age^20–22^, male sex^21–23^, presence of comorbid diseases^20,21^ were associated with increased odds of in-hospital mortality. In addition, a meta-analysis revealed that cardiovascular disease, pulmonary disease, and diabetes significantly increased the risk of mortality after hip fracture surgery^26^. Heart failure, renal failure, pulmonary disease were contributive factors for in-hospital mortality among hip fracture patients after surgery^22^. A small retrospective study illustrated that type of fracture and type of treatment were associated with 1-year mortality after fragility hip fracture^24^. Our study also newly found that fracture type and operation type also impact the in-hospital mortality, and it suggested that preoperative surgical plan should be carefully made.

### Prediction of in-hospital mortality among hip fracture

Several studies have utilized machine learning algorithms to predict mortality in patients with hip fractures (Table 3), considering factors such as age, comorbidities, functional status, and laboratory markers. Forssten *et al*.^25^ developed a machine learning model and achieved an AUC of 0.74 for predicting 1-year mortality in a cohort of 124 707 hip fracture surgery patients. They also developed another model for predicting 30-day mortality, with an AUC of 0.76^27^. Oosterhoff *et al*.^28^ proposed a machine learning model for predicting 90-day and 2-year mortality in 2478 femoral neck fracture patients aged 65 or above. They obtained a c-statistic of 0.74 for 90-day mortality and 0.70 for 2-year mortality prediction. In a prospective study by Dijkstra *et al*.^29^, machine learning models were established to predict 90-day and 1-year mortality in elderly patients with femoral neck fractures, achieving c-statistics of 0.80 and 0.76, respectively. Our team previously developed a machine learning-based model for predicting in-hospital mortality, specifically in critically ill hip fracture patients^30^. The model included parameters such as age, sex, anemia, mechanical ventilation, cardiac arrest, and chronic airway obstruction, with an AUC of 0.715^30^. A meta-analysis demonstrated that machine learning models outperformed the main clinical scale (Nottingham Hip Fracture Score, C-index: 0.702) with a pooled C-index of 0.762 in the training set and 0.838 in the validation set^5^. This study concludes by suggesting further research to improve predictive performance by incorporating machine learning models into clinical scoring systems.

However, most of these models were limited to specific populations or single-center studies with smaller sample sizes. In addition, the prediction models were mainly designed to predict the 30-day, 90-day, or 1-year mortality among hip fracture patients^24,25,28,29,31–35^. Furthermore, the prediction performance of these models needs improvements. Our study, on the other hand, utilized a national cohort of a large sample size, allowing for more generalizability of the results. Notably, our comprehensive scoring system incorporating multiple evaluation metrics provides a robust assessment of the prediction performance, allowing for a more accurate comparison between the models. The AUC of our eXGBM model was up to 0.908, indicating favorable prediction performance. The superiority of our eXGBM model can be attributed to its ability to handle complex interactions and nonlinear relationships within the data, thereby capturing important predictive patterns. The high AUC, accuracy, precision, specificity, and F1 score of the eXGBM model also demonstrated its excellent discrimination and predictive capability. Additionally, the high score achieved by the eXGBM model in our comprehensive evaluation system further confirms its superior performance compared to other models.

### The epidemiology of in-hospital mortality among hip fracture

Our findings revealed a postoperative in-hospital mortality rate of 0.9%, which is consistent with previous studies reported in the literature. Previous studies showed similar mortality rates ranging from 1.8 to 3.0%^22,36,37^. Nonetheless, some studies reported a relatively high in-hospital mortality among hip fracture patients. For instance, Peterle *et al*.^38^ illustrated that the mean hospital mortality rate was 18.4% after analyzing 402 patients over 60 years of age admitted to hospital due to osteoporotic hip fracture. Sanz-Reig *et al*.^39^ reported that the in-hospital mortality rate was 11.4% in a prospective study conducted on 311 hip fracture patients with an age of more than 65 years. The variation in in-hospital mortality could be explained by the heterogeneity of populations. The relatively low in-hospital mortality rate observed in our study can be attributed to several factors. Firstly, the inclusion of geriatric hip fracture patients from multiple hospitals across the nation provides a representative sample, reducing selection bias. Additionally, improvements in surgical techniques, perioperative care, and rehabilitation protocols have contributed to better patient outcomes and decreased mortality rates over the years^40^. It is also possible that the exclusion of patients with severe comorbidities or high-risk surgical candidates in our study might have influenced the mortality rate.

### Individualized management for geriatric hip fracture

The implementation of our developed AI model using the eXGBM technique holds significant clinical implications. Accurate risk prediction of postoperative in-hospital mortality can guide clinicians in optimizing treatment strategies, such as tailored surgical approaches, specific anesthesia plans, and early mobilization protocols, to improve patient outcomes. Moreover, identification of high-risk patients allows for appropriate allocation of healthcare resources and timely intervention to reduce mortality rates. Individualized management can be achieved. For geriatric hip fracture patients identified as high-risk for postoperative in-hospital mortality, a proactive and individualized management approach is essential. Close monitoring of vital signs and regular pain assessment should be implemented to promptly identify any signs of deterioration. Early intervention by a multidisciplinary team, comprehensive medication management, and optimization are crucial to address potential complications and minimize adverse drug events. Specialized rehabilitation programs tailored to their specific needs and comorbidities can optimize functional recovery. Postoperative infection prevention strategies and regular communication among healthcare providers ensure seamless coordination and continuity of care. Geriatric hip fracture patients identified as low-risk for postoperative in-hospital mortality also require comprehensive postoperative management. Standard care protocols such as pain management, wound care, and mobilization should be followed consistently. Early mobilization protocols and physical therapy should be integrated into their care plan to facilitate optimal recovery and functional outcomes. Adequate nutrition support, psychological support, and regular follow-up appointments are important for healing and well-being. Patient education and engagement in the care plan ensure a clear understanding of postoperative instructions and adherence to medication regimens, contributing to a smooth transition to a home setting.

### Limitations

Despite the promising findings of our study, there are several limitations that should be acknowledged. Firstly, our study focused on predicting in-hospital mortality and does not provide insights into long-term outcomes or mortality after discharge. Future studies should address this limitation to provide a comprehensive understanding of the overall prognosis of geriatric hip fracture patients. Secondly, the variables included in our model were limited to those available in the dataset, and there might be other important predictors not included in our analysis. Incorporation of additional variables, such as frailty indices or surgical complications, could further improve the predictive accuracy of the model. Lastly, our study was retrospective in nature, which might pose challenges in determining the causal relationship between variables. Thus, further research is needed to overcome these limitations and enhance our understanding of the factors influencing the prognosis of geriatric hip fracture patients. Additionally, the use of interventional studies and larger, more diverse datasets could help to validate and refine the predictive models, ultimately improving the accuracy and clinical utility of these models for geriatric hip fracture patients.

## Conclusions

In conclusion, our study successfully develops an AI model to predict the risk of postoperative in-hospital mortality in geriatric hip fracture patients. The eXGBM model demonstrates excellent predictive performance, outperforming other models in terms of discrimination and calibration. The implementation of this model in clinical practice could enhance risk stratification and aid clinicians in making informed treatment decisions to optimize patient outcomes and resource allocation in the management of geriatric hip fractures. Further research is warranted to address the limitations and explore the long-term prognostic implications of our AI model.

## Ethical approval

This study was approved by the Ethics Committee of the Chinese PLA General Hospital (Judgement’s reference number: S2019-014-01). All data were analyzed anonymously, and this study abided by the Helsinki Declaration.

## Consent

Written informed consent was obtained from the patient for publication and any accompanying images. A copy of the written consent is available for review by the Editor-in-Chief of this journal on request.

## Source of funding

This study was funded by National Clinical Research Center for Orthopedics, Sports Medicine and Rehabilitation of China and National Key Research and Development Program of China (No.2022YFC2504300 and No.2022YFC2504301). This study was also funded by Hainan Province Clinical Medical Center and Military Training Injury Prevention and Control Special Project (No. 21XLS38).

## Author contribution

All authors contributed to the study design, conducted the data collection and analyses, and drafted the paper. All authors have read and approved the manuscript.

## Conflicts of interest disclosure

The authors declare that they have no financial conflict of interest with regard to the content of this report.

## Research registration unique identifying number (UIN)

1. Name of the registry: ChiCTR: Chinese Clinical Trial Registry.
2. Unique identifying number or registration ID: ChiCTR2000031423.
3. Hyperlink to your specific registration (must be publicly accessible and will be checked): https://www.chictr.org.cn/showproj.html?proj=51335.

## Guarantor

Peifu Tang, Licheng Zhang, and Pengbin Yin.

## Data availability statement

The datasets of the current study are available under reasonable request.

## Provenance and peer review

Not commissioned, externally peer-reviewed.

## Supplementary Material

**Figure:**
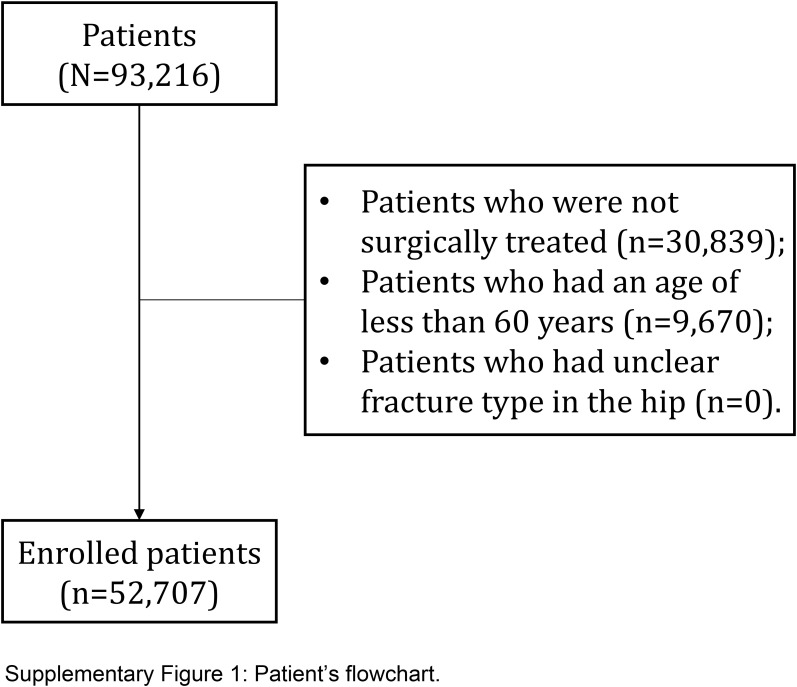


**Figure:**
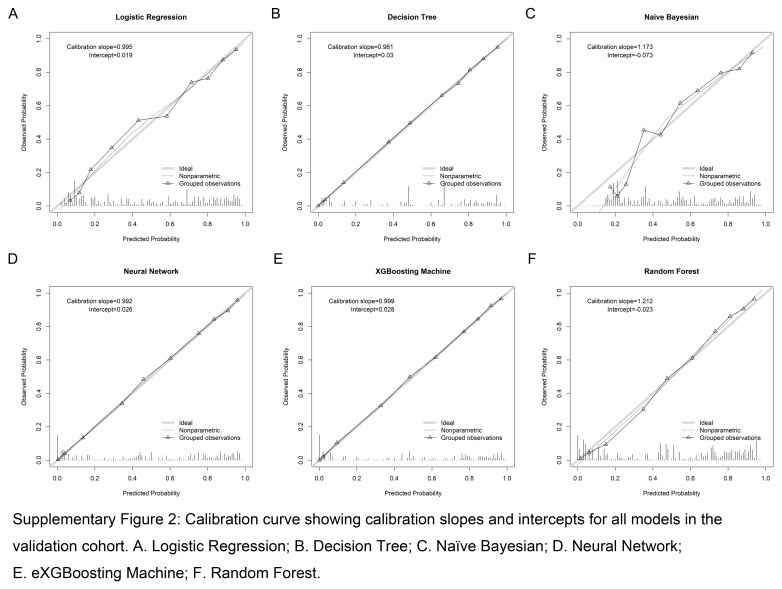


**Figure:**
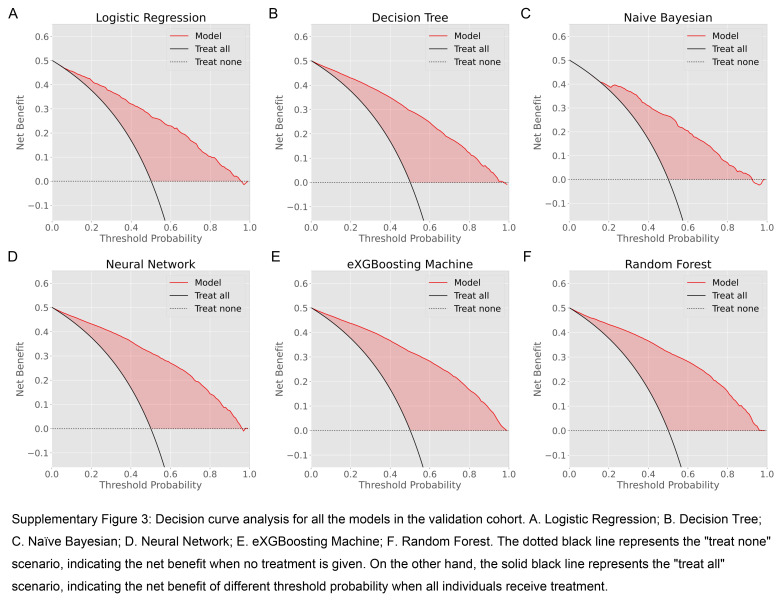


**Figure:**
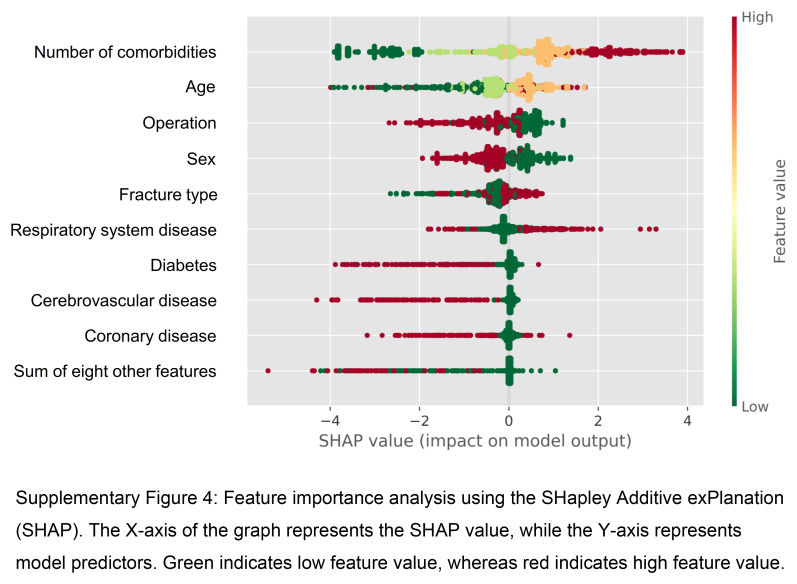


**Figure:**
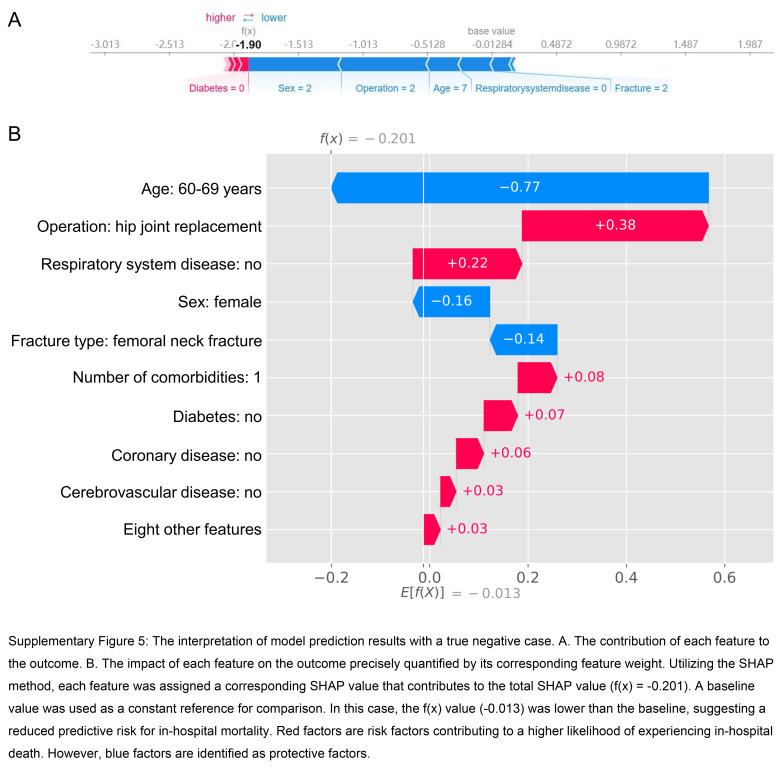


**Figure:**
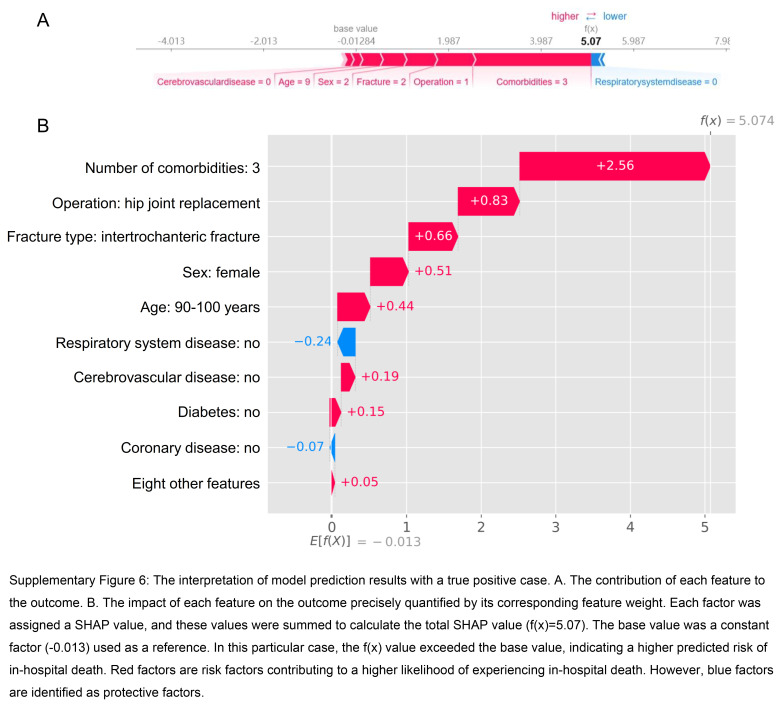


## References

[1] Griffiths R Babu S Dixon P . Guideline for the management of hip fractures 2020: guideline by the association of anaesthetists. Anaesthesia 2021;76:225–237. 33289066 10.1111/anae.15291

[2] Dong Y Zhang Y Song K . What was the epidemiology and global burden of disease of hip fractures from 1990 to 2019? Results from and additional analysis of the global burden of disease study 2019. Clin Orthop Relat Res 2023;481:1209–1220. 36374576 10.1097/CORR.0000000000002465 PMC10194687

[3] Sing CW Lin TC Bartholomew S . Global epidemiology of hip fractures: secular trends in incidence rate, post-fracture treatment, and all-cause mortality. J Bone Miner Res 2023;38:1064–75. 37118993 10.1002/jbmr.4821

[4] Lex JR Di Michele J Koucheki R . Artificial intelligence for hip fracture detection and outcome prediction: a systematic review and meta-analysis. JAMA Netw Open 2023;6:e233391. 36930153 10.1001/jamanetworkopen.2023.3391 PMC10024206

[5] Liu F Liu C Tang X . Predictive value of machine learning models in postoperative mortality of older adults patients with hip fracture: a systematic review and meta-analysis. Arch Gerontol Geriatr 2023;115:105120. 37473692 10.1016/j.archger.2023.105120

[6] Lei M Han Z Wang S . Biological signatures and prediction of an immunosuppressive status-persistent critical illness-among orthopedic trauma patients using machine learning techniques. Front Immunol 2022;13:979877. 36325351 10.3389/fimmu.2022.979877 PMC9620964

[7] Gao L Cao Y Cao X . Machine learning-based algorithms to predict severe psychological distress among cancer patients with spinal metastatic disease. The Spine Journal 2023;S1529-9430:00199–00197. 10.1016/j.spinee.2023.05.009 37182703

[8] Cui Y Shi X Wang S . Machine learning approaches for prediction of early death among lung cancer patients with bone metastases using routine clinical characteristics: an analysis of 19,887 patients. Front Public Health 2022;10:1019168. 36276398 10.3389/fpubh.2022.1019168 PMC9583680

[9] Murphy EA Ehrhardt B Gregson CL . Machine learning outperforms clinical experts in classification of hip fractures. Sci Rep 2022;12:2058. 35136091 10.1038/s41598-022-06018-9 PMC8825848

[10] Shi X Cui Y Wang S . Development and validation of a web-based artificial intelligence prediction model to assess massive intraoperative blood loss for metastatic spinal disease using machine learning techniques. Spine J 2024;24:146–160. 37704048 10.1016/j.spinee.2023.09.001

[11] Mathew G Agha R Albrecht J . STROCSS 2021: strengthening the reporting of cohort, cross-sectional and case-control studies in surgery. Int J Surg 2021;96:106165. 34774726 10.1016/j.ijsu.2021.106165

[12] Collins GS Reitsma JB Altman DG . Transparent reporting of a multivariable prediction model for individual prognosis or diagnosis (TRIPOD). Ann Intern Med 2015;162:735–736. 10.7326/L15-5093-2 25984857

[13] Liu X Guo L Wang H . Research on imbalance machine learning methods for MR[Formula: see text]WI soft tissue sarcoma data. BMC Med Imaging 2022;22:149. 36028803 10.1186/s12880-022-00876-5 PMC9417078

[14] Zhu C Xu Z Gu Y . Prediction of post-stroke urinary tract infection risk in immobile patients using machine learning: an observational cohort study. J Hosp Infect 2022;122:96–107. 35045341 10.1016/j.jhin.2022.01.002

[15] Johnson AE Pollard TJ Shen L . MIMIC-III, a freely accessible critical care database. Sci Data 2016;3:160035. 27219127 10.1038/sdata.2016.35 PMC4878278

[16] Hu C Li L Huang W . Interpretable machine learning for early prediction of prognosis in sepsis: a discovery and validation study. Infect Dis Ther 2022;11:1117–1132. 35399146 10.1007/s40121-022-00628-6 PMC9124279

[17] Wang K Tian J Zheng C . Interpretable prediction of 3-year all-cause mortality in patients with heart failure caused by coronary heart disease based on machine learning and SHAP. Comput Biol Med 2021;137:104813. 34481185 10.1016/j.compbiomed.2021.104813

[18] Lundberg SM Nair B Vavilala MS . Explainable machine-learning predictions for the prevention of hypoxaemia during surgery. Nat Biomed Eng 2018;2:749–760. 31001455 10.1038/s41551-018-0304-0 PMC6467492

[19] Xiong F Cao X Shi X . A machine learning-based model to predict early death among bone metastatic breast cancer patients: a large cohort of 16,189 patients. Front Cell Dev Biol 2022;10:1059597. 36568969 10.3389/fcell.2022.1059597 PMC9768487

[20] Pande I Scott DL O’Neill TW . Quality of life, morbidity, and mortality after low trauma hip fracture in men. Ann Rheum Dis 2006;65:87–92. 16079173 10.1136/ard.2004.034611 PMC1797995

[21] Chatterton BD Moores TS Ahmad S . Cause of death and factors associated with early in-hospital mortality after hip fracture. Bone Joint J 2015;97-B:246–251. 25628290 10.1302/0301-620X.97B2.35248

[22] Endo A Baer HJ Nagao M . Prediction model of in-hospital mortality after hip fracture surgery. Journal of orthopaedic trauma 2018;32:34–38. 29076984 10.1097/BOT.0000000000001026

[23] Katsoulis M Benetou V Karapetyan T . Excess mortality after hip fracture in elderly persons from Europe and the USA: the CHANCES project. J Intern Med 2017;281:300–310. 28093824 10.1111/joim.12586

[24] Kitcharanant N Chotiyarnwong P Tanphiriyakun T . Development and internal validation of a machine-learning-developed model for predicting 1-year mortality after fragility hip fracture. BMC Geriatr 2022;22:451. 35610589 10.1186/s12877-022-03152-x PMC9131628

[25] Forssten MP Bass GA Ismail AM . Predicting 1-year mortality after hip fracture surgery: an evaluation of multiple machine learning approaches. J Pers Med 2021;11:727. 34442370 10.3390/jpm11080727 PMC8401745

[26] Chang W Lv H Feng C . Preventable risk factors of mortality after hip fracture surgery: Systematic review and meta-analysis. Int J Surg 2018;52:320–328. 29530826 10.1016/j.ijsu.2018.02.061

[27] Cao Y Forssten MP Mohammad Ismail A . Predictive values of preoperative characteristics for 30-day mortality in traumatic hip fracture patients. J Pers Med 2021;11:353. 33924993 10.3390/jpm11050353 PMC8146802

[28] Oosterhoff JHF Savelberg A Karhade AV . Development and internal validation of a clinical prediction model using machine learning algorithms for 90 day and 2 year mortality in femoral neck fracture patients aged 65 years or above. Eur J Trauma Emerg Surg 2022;48:4669–4682. 35643788 10.1007/s00068-022-01981-4 PMC9712294

[29] Dijkstra H Oosterhoff JHF van de Kuit A . Development of machine-learning algorithms for 90-day and one-year mortality prediction in the elderly with femoral neck fractures based on the HEALTH and FAITH trials. Bone Jt Open 2023;4:168–181. 37051847 10.1302/2633-1462.43.BJO-2022-0162.R1 PMC10032237

[30] Lei M Han Z Wang S . A machine learning-based prediction model for in-hospital mortality among critically ill patients with hip fracture: an internal and external validated study. Injury 2023;54:636–644. 36414503 10.1016/j.injury.2022.11.031

[31] Hjelholt TJ Johnsen SP Brynningsen PK . Development and validation of a model for predicting mortality in patients with hip fracture. Age Ageing 2022;51:afab233. 34923589 10.1093/ageing/afab233

[32] Zhang Y Huang L Liu Y . Prediction of mortality at one year after surgery for pertrochanteric fracture in the elderly via a Bayesian belief network. Injury 2020;51:407–413. 31870611 10.1016/j.injury.2019.11.029

[33] DeBaun MR Chavez G Fithian A . Artificial neural networks predict 30-day mortality after hip fracture: insights from machine learning. J Am Acad Orthop Surg 2021;29:977–983. 33315645 10.5435/JAAOS-D-20-00429

[34] Li Y Chen M Lv H . A novel machine-learning algorithm for predicting mortality risk after hip fracture surgery. Injury 2021;52:1487–1493. 33386157 10.1016/j.injury.2020.12.008

[35] Cary MP Jr Zhuang F Draelos RL . Machine learning algorithms to predict mortality and allocate palliative care for older patients with hip fracture. J Am Med Dir Assoc 2021;22:291–296. 33132014 10.1016/j.jamda.2020.09.025 PMC7867606

[36] Yoo S Jang EJ Jo J . The association between hospital case volume and in-hospital and one-year mortality after hip fracture surgery. The Bone & Joint Journal 2020;102-B:1384–1391. 32993327 10.1302/0301-620X.102B10.BJJ-2019-1728.R3

[37] Groff H Kheir MM George J . Causes of in-hospital mortality after hip fractures in the elderly. Hip Int 2020;30:204–209. 30909746 10.1177/1120700019835160

[38] Peterle VCU Novaes M Junior PEB . Osteoporotic hip fracture-Comorbidities and factors associated with in-hospital mortality in the elderly: a nine-year cohort study in Brazil. PLoS One 2022;17:e0272006. 35960782 10.1371/journal.pone.0272006 PMC9374234

[39] Sanz-Reig J Salvador Marin J Ferrandez Martinez J . Prognostic factors and predictive model for in-hospital mortality following hip fractures in the elderly. Chin J Traumatol 2018;21:163–169. 29784590 10.1016/j.cjtee.2017.10.006 PMC6033736

[40] Trevisan C Gallinari G Klumpp R . Year to year comparison of 2000-2015 in hip fracture management: same survival rate despite older and more fragile patients. Aging Clin Exp Res 2019;31:1097–1103. 30276632 10.1007/s40520-018-1047-1

