# Supplementary material for: Establishment and validation of an artificial intelligence web application for predicting postoperative in-hospital mortality in patients with hip fracture: a national cohort study of 52 707 cases
Source: Int J Surg. 2024 May 15;110(8):4876–92. doi: 10.1097/JS9.0000000000001599 (PMC11325965; doi:10.1097/JS9.0000000000001599)
Supplement: Supplementary file 2 [file js9-110-4876-s003.docx]

**Supplementary File 1**

**Methods for external validation**

This study extracted data from the Medical Information Mart for Intensive Care III (MIMIC-III) database, and the data on patients with hip fracture were collected. The MIMIC-III database was constructed by the XXX (The removal of affiliations due to the double-blind peer review policy) in another country. This database is an excellent resource for external validation of our research because it contains a vast amount of comprehensive, longitudinal clinical data on intensive care unit (ICU) patients, including information on demographics, comorbidities, laboratory tests, and vital signs. This wealth of data allows for robust variable comparison, such as the basic information and co-morbidity information that is central to our study. By validating our model’s performance on data from this diverse and well-established database, we can strengthen its generalizability and clinical relevance, ensuring that our research findings have a broad and meaningful impact.

The use of the MIMIC-III database in this study is guided by the ethical principles of respect for patient privacy and autonomy, transparency, and responsible data stewardship. The MIMIC-III database is a comprehensive, longitudinal database containing clinical data from intensive care unit (ICU) patients, which has been de-identified to protect patient privacy. We ensure that our research adheres to the highest ethical standards by anonymously analyzing the data and strictly adhering to the terms of use specified by the database administrators.

Access to the MIMIC-III database is granted after completing the National Institutes of Health’s web-based training course on Protecting Human Research Participants and the Good Clinical Practice course. After completing these requirements, researchers can request access to the database through the Institute of Medical Engineering and Science at the XXX (The removal of affiliations due to the double-blind peer review policy) [1]. Once approved, researchers can access and download the de-identified data for their research studies. The use of such a comprehensive and well-curated database like MIMIC-III enables researchers to conduct high-quality studies. Since the data in the database has been de-identified, patient consent was not required. We commit to strictly adhere to ethical guidelines and legal regulations at all stages of the research and ensure the appropriate use and protection of patient privacy.

As a result, 394 patients with hip fracture were collected. Patient’s flowchart is depicted in **Figure 1** in this file. We excluded those who were not treated with surgery for hip fracture (n=69), those who had an age of less than 60 years (n=78), and those who had unclear fracture type in the hip (n=1). Thus, based on the same inclusive and exclusive criteria in the study, a series of 246 patients were included for analysis as an external validation cohort.


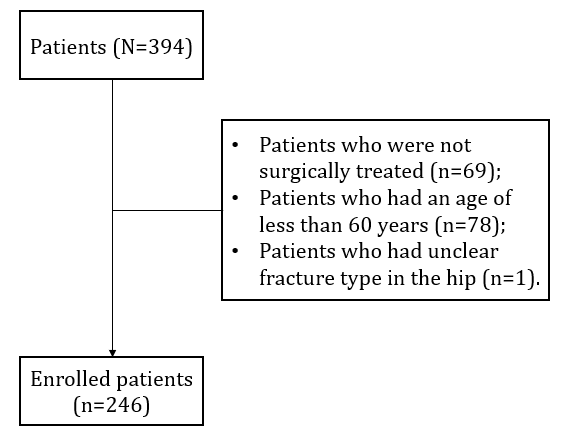


**Figure 1:** Patient’s flowchart.

**Results for external validation**

Based on the same inclusive and exclusive criteria in the study, a series of 246 patients were included for analysis as an external validation cohort. The baseline characteristics are summarized in **Table 1** in this file. The area under the curve (AUC) value of the model was 0.784 (95%CI: 0.699-0.869) in the external validation cohort (**Figure 2** in this file), indicating relatively favorable prediction even in the ICU settings. In addition, the accuracy was 0.907, Brier score was 0.095, and Log loss was 0.464. After applying 1000 bootstrap, the calibration curve demonstrated that the eXGBM model also had favorable calibration (**Figure 3** in this file). The model in our study was established based on patients in the general ward, but it still achieved a favorable AUC value in the ICU population. This indicated that the model also had a good extrapolation effect and could predict outcomes effectively across different patient populations, showcasing its robustness and clinical relevance.


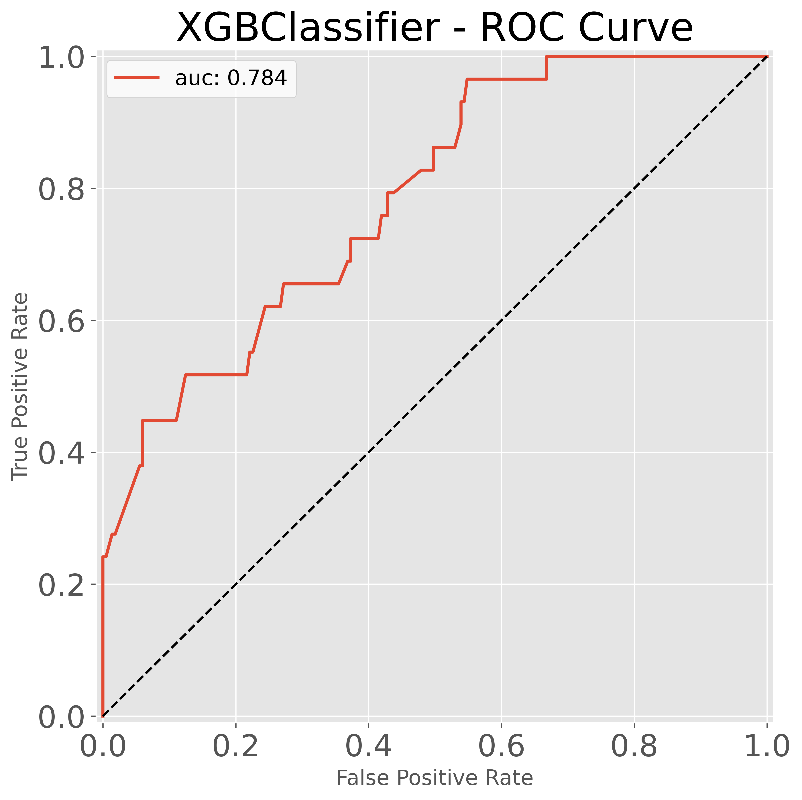


**Figure 2:** The AUC value in the external validation cohort.


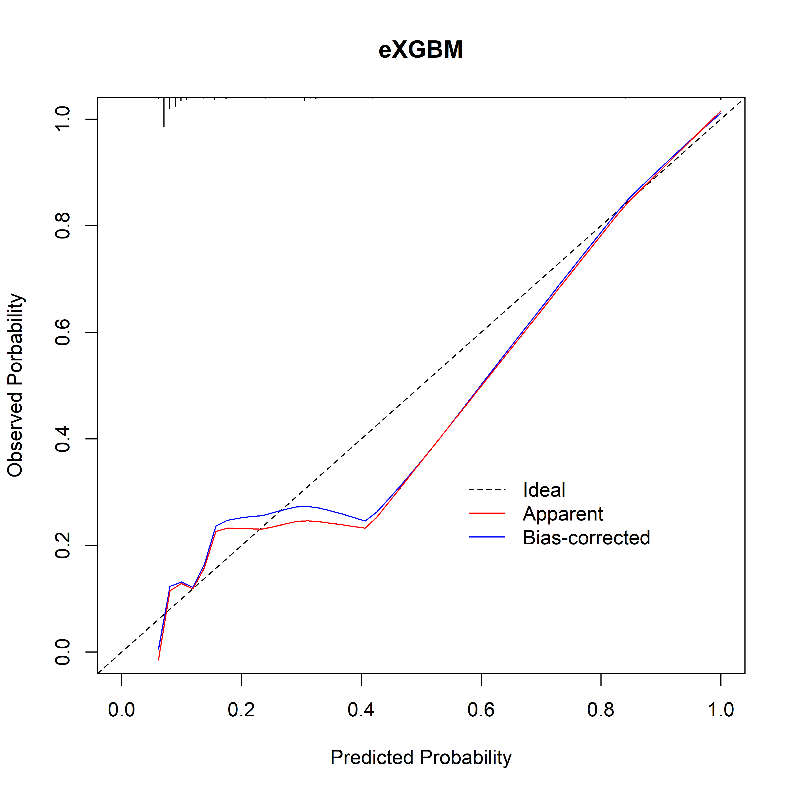


**Figure 2:** The calibration curve in the external validation cohort. eXGBM, extreme gradient boosting machine.

| **Table 1.** Baseline characteristics of the external validation cohort. | |
| --- | --- |
| Clinical characteristics | Overall |
| n | 246 |
| Age (years, mean (SD)) | 80.58 (8.08) |
| Sex (male/female, %) | 100/146 (40.7/59.3) |
| Fracture type (femoral neck fracture/intertrochanteric fracture, %) | 96/150 (39.0/61.0) |
| Operation (Hip joint replacement/Internal fixation, %) | 78/168 (31.7/68.3) |
| Comorbidities (%) |  |
| 0 | 4 (1.6) |
| 1 | 27 (11.0) |
| 2 | 65 (26.4) |
| ≧3 | 150 (61.0) |
| Coronary disease (no/yes, %) | 190/56 (77.2/22.8) |
| Cerebrovascular disease (no/yes, %) | 17/229 (6.9/93.1) |
| Heart failure (no/yes, %) | 140/106 (56.9/43.1) |
| Renal failure (no/yes, %) | 236/10 (95.9/4.1) |
| Nephrotic syndrome (no/yes, %) | 241/5 (98.0/2.0) |
| Respiratory system disease (no/yes, %) | 106/140 (43.1/56.9) |
| Gastrointestinal bleeding (no/yes, %) | 235/11 (95.5/4.5) |
| Gastrointestinal ulcer (no/yes, %) | 246/0 (100.0/0.0) |
| Liver failure (no/yes, %) | 246/0 (100.0/0.0) |
| Cirrhosis (no/yes, %) | 237/9 (96.3/3.7) |
| Diabetes (no/yes, %) | 172/74 (69.9/30.1) |
| Cancer (no/yes, %) | 206/40 (83.7/16.3) |
| Death in hospital (no/yes, %) | 217/29 (88.2/11.8) |
| SD, standard deviation. | |

**References**

1. Johnson AE, Pollard TJ, Shen L, Lehman LW, Feng M, Ghassemi M, Moody B, Szolovits P, Celi LA, Mark RG: MIMIC-III, a freely accessible critical care database. *Sci Data* 2016, 3:160035.
