# Supplementary material for: Establishment and validation of an artificial intelligence web application for predicting postoperative in-hospital mortality in patients with hip fracture: a national cohort study of 52 707 cases
Source: Int J Surg. 2024 May 15;110(8):4876–92. doi: 10.1097/JS9.0000000000001599 (PMC11325965; doi:10.1097/JS9.0000000000001599)
Supplement: Supplementary file 3 [file js9-110-4876-s004.docx]

| **Supplementary Table 1**. Subgroup analysis of clinical characteristics according to the age. | | | | | | | |
| --- | --- | --- | --- | --- | --- | --- | --- |
| Characteristics | Overall | Age (years) | | | | | p |
|  |  | 60-69 | 70-79 | 80-89 | 90-100 | >100 |  |
| n | 52707 | 13285 | 23093 | 14520 | 1753 | 56 |  |
| Sex (male/female, %) | 19200/33507 (36.4/63.6) | 5438/7847 (40.9/59.1) | 8380/14713 (36.3/63.7) | 4851/9669 (33.4/66.6) | 517/1236 (29.5/70.5) | 14/42 (25.0/75.0) | <0.001 |
| Fracture type (femoral neck fracture/intertrochanteric fracture, %) | 30251/22456 (57.4/42.6) | 8903/4382 (67.0/33.0) | 13308/9785 (57.6/42.4) | 7247/7273 (49.9/50.1) | 767/986 (43.8/56.2) | 26/30 (46.4/53.6) | <0.001 |
| Operation (Hip joint replacement/Internal fixation, %) | 27274/25433 (51.7/48.3) | 7040/6245 (53.0/47.0) | 12126/10967 (52.5/47.5) | 7242/7278 (49.9/50.1) | 840/913 (47.9/52.1) | 26/30 (46.4/53.6) | <0.001 |
| Number of comorbidities (%) |  |  |  |  |  |  | <0.001 |
| 0 | 25904 (49.1) | 7580 (57.1) | 11084 (48.0) | 6419 (44.2) | 792 (45.2) | 29 (51.8) |  |
| 1 | 12299 (23.3) | 3011 (22.7) | 5399 (23.4) | 3479 (24.0) | 400 (22.8) | 10 (17.9) |  |
| 2 | 11933 (22.6) | 2219 (16.7) | 5411 (23.4) | 3834 (26.4) | 455 (26.0) | 14 (25.0) |  |
| ≧3 | 2571 (4.9) | 475 (3.6) | 1199 (5.2) | 788 (5.4) | 106 (6.0) | 3 (5.4) |  |
| Anemia (no/yes, %) | 52362/345 (99.3/0.7) | 13234/51 (99.6/0.4) | 22964/129 (99.4/0.6) | 14386/134 (99.1/0.9) | 1722/31 (98.2/1.8) | 56/0 (100.0/0.0) | <0.001 |
| Hypertension (no/yes, %) | 47865/4842 (90.8/9.2) | 12386/899 (93.2/6.8) | 20768/2325 (89.9/10.1) | 13060/1460 (89.9/10.1) | 1599/154 (91.2/8.8) | 52/4 (92.9/7.1) | <0.001 |
| Coronary disease (no/yes, %) | 50865/1842 (96.5/3.5) | 13036/249 (98.1/1.9) | 22202/891 (96.1/3.9) | 13884/636 (95.6/4.4) | 1689/64 (96.3/3.7) | 54/2 (96.4/3.6) | <0.001 |
| Cerebrovascular disease (no/yes, %) | 50987/1720 (96.7/3.3) | 12897/388 (97.1/2.9) | 22249/844 (96.3/3.7) | 14065/455 (96.9/3.1) | 1721/32 (98.2/1.8) | 55/1 (98.2/1.8) | <0.001 |
| Heart failure (no/yes, %) | 52541/166 (99.7/0.3) | 13268/17 (99.9/0.1) | 23036/57 (99.8/0.2) | 14441/79 (99.5/0.5) | 1741/12 (99.3/0.7) | 55/1 (98.2/1.8) | <0.001 |
| Atherosclerosis (no/yes, %) | 52410/297 (99.4/0.6) | 13220/65 (99.5/0.5) | 22966/127 (99.5/0.5) | 14427/93 (99.4/0.6) | 1742/11 (99.4/0.6) | 55/1 (98.2/1.8) | 0.338 |
| Renal failure (no/yes, %) | 52514/193 (99.6/0.4) | 13251/34 (99.7/0.3) | 23002/91 (99.6/0.4) | 14461/59 (99.6/0.4) | 1745/8 (99.5/0.5) | 55/1 (98.2/1.8) | 0.06 |
| Nephrotic syndrome (no/yes, %) | 52701/6 (100.0/0.0) | 13284/1 (100.0/0.0) | 23090/3 (100.0/0.0) | 14518/2 (100.0/0.0) | 1753/0 (100.0/0.0) | 56/0 (100.0/0.0) | 0.973 |
| Respiratory system disease (no/yes, %) | 49216/3491 (93.4/6.6) | 12846/439 (96.7/3.3) | 21667/1426 (93.8/6.2) | 13136/1384 (90.5/9.5) | 1517/236 (86.5/13.5) | 50/6 (89.3/10.7) | <0.001 |
| Gastrointestinal bleeding (no/yes, %) | 52651/56 (99.9/0.1) | 13279/6 (100.0/0.0) | 23072/21 (99.9/0.1) | 14495/25 (99.8/0.2) | 1749/4 (99.8/0.2) | 56/0 (100.0/0.0) | 0.009 |
| Gastrointestinal ulcer (no/yes, %) | 52637/70 (99.9/0.1) | 13272/13 (99.9/0.1) | 23067/26 (99.9/0.1) | 14492/28 (99.8/0.2) | 1750/3 (99.8/0.2) | 56/0 (100.0/0.0) | 0.188 |
| Liver failure (no/yes, %) | 52703/4 (100.0/0.0) | 13285/0 (100.0/0.0) | 23091/2 (100.0/0.0) | 14518/2 (100.0/0.0) | 1753/0 (100.0/0.0) | 56/0 (100.0/0.0) | 0.752 |
| Cirrhosis (no/yes, %) | 52592/115 (99.8/0.2) | 13255/30 (99.8/0.2) | 23031/62 (99.7/0.3) | 14498/22 (99.8/0.2) | 1752/1 (99.9/0.1) | 56/0 (100.0/0.0) | 0.095 |
| Gastritis (no/yes, %) | 52652/55 (99.9/0.1) | 13276/9 (99.9/0.1) | 23070/23 (99.9/0.1) | 14501/19 (99.9/0.1) | 1749/4 (99.8/0.2) | 56/0 (100.0/0.0) | 0.251 |
| Diabetes (no/yes, %) | 49613/3094 (94.1/5.9) | 12442/843 (93.7/6.3) | 21589/1504 (93.5/6.5) | 13832/688 (95.3/4.7) | 1694/59 (96.6/3.4) | 56/0 (100.0/0.0) | <0.001 |
| Dementia (no/yes, %) | 52515/192 (99.6/0.4) | 13265/20 (99.8/0.2) | 23006/87 (99.6/0.4) | 14445/75 (99.5/0.5) | 1744/9 (99.5/0.5) | 55/1 (98.2/1.8) | <0.001 |
| Cancer (no/yes, %) | 52117/590 (98.9/1.1) | 13154/131 (99.0/1.0) | 22813/280 (98.8/1.2) | 14360/160 (98.9/1.1) | 1735/18 (99.0/1.0) | 55/1 (98.2/1.8) | 0.362 |
| Death in hospital (no/yes, %) | 52257/450 (99.1/0.9) | 13241/44 (99.7/0.3) | 22932/161 (99.3/0.7) | 14325/195 (98.7/1.3) | 1703/50 (97.1/2.9) | 56/0 (100.0/0.0) | <0.001 |
