# Supplementary material for: Establishment and validation of an artificial intelligence web application for predicting postoperative in-hospital mortality in patients with hip fracture: a national cohort study of 52 707 cases
Source: Int J Surg. 2024 May 15;110(8):4876–92. doi: 10.1097/JS9.0000000000001599 (PMC11325965; doi:10.1097/JS9.0000000000001599)
Supplement: Supplementary file 4 [file js9-110-4876-s005.docx]

| **Supplementary Table 2**. Subgroup analysis of clinical characteristics according to sex. | | | | |
| --- | --- | --- | --- | --- |
| Characteristics | Overall | Sex | | p |
|  |  | Male | Female |  |
| n | 52707 | 19200 | 33507 |  |
| Age (%) |  |  |  | <0.001 |
| 60-69 | 13285 (25.2) | 5438 (28.3) | 7847 (23.4) |  |
| 70-79 | 23093 (43.8) | 8380 (43.6) | 14713 (43.9) |  |
| 80-89 | 14520 (27.5) | 4851 (25.3) | 9669 (28.9) |  |
| 90-100 | 1753 (3.3) | 517 (2.7) | 1236 (3.7) |  |
| >100 | 56 (0.1) | 14 (0.1) | 42 (0.1) |  |
| Fracture type (femoral neck fracture/intertrochanteric fracture, %) | 30251/22456 (57.4/42.6) | 9775/9425 (50.9/49.1) | 20476/13031 (61.1/38.9) | <0.001 |
| Operation (Hip joint replacement/Internal fixation, %) | 27274/25433 (51.7/48.3) | 8622/10578 (44.9/55.1) | 18652/14855 (55.7/44.3) | <0.001 |
| Number of comorbidities (%) |  |  |  | <0.001 |
| 0 | 25904 (49.1) | 9268 (48.3) | 16636 (49.6) |  |
| 1 | 12299 (23.3) | 4414 (23.0) | 7885 (23.5) |  |
| 2 | 11933 (22.6) | 4433 (23.1) | 7500 (22.4) |  |
| ≧3 | 2571 (4.9) | 1085 (5.7) | 1486 (4.4) |  |
| Anemia (no/yes, %) | 52362/345 (99.3/0.7) | 19101/99 (99.5/0.5) | 33261/246 (99.3/0.7) | 0.003 |
| Hypertension (no/yes, %) | 47865/4842 (90.8/9.2) | 17618/1582 (91.8/8.2) | 30247/3260 (90.3/9.7) | <0.001 |
| Coronary disease (no/yes, %) | 50865/1842 (96.5/3.5) | 18577/623 (96.8/3.2) | 32288/1219 (96.4/3.6) | 0.019 |
| Cerebrovascular disease (no/yes, %) | 50987/1720 (96.7/3.3) | 18368/832 (95.7/4.3) | 32619/888 (97.3/2.7) | <0.001 |
| Heart failure (no/yes, %) | 52541/166 (99.7/0.3) | 19138/62 (99.7/0.3) | 33403/104 (99.7/0.3) | 0.868 |
| Atherosclerosis (no/yes, %) | 52410/297 (99.4/0.6) | 19075/125 (99.3/0.7) | 33335/172 (99.5/0.5) | 0.049 |
| Renal failure (no/yes, %) | 52514/193 (99.6/0.4) | 19106/94 (99.5/0.5) | 33408/99 (99.7/0.3) | 0.001 |
| Nephrotic syndrome (no/yes, %) | 52701/6 (100.0/0.0) | 19195/5 (100.0/0.0) | 33506/1 (100.0/0.0) | 0.05 |
| Respiratory system disease (no/yes, %) | 49216/3491 (93.4/6.6) | 17421/1779 (90.7/9.3) | 31795/1712 (94.9/5.1) | <0.001 |
| Gastrointestinal bleeding (no/yes, %) | 52651/56 (99.9/0.1) | 19169/31 (99.8/0.2) | 33482/25 (99.9/0.1) | 0.005 |
| Gastrointestinal ulcer (no/yes, %) | 52637/70 (99.9/0.1) | 19169/31 (99.8/0.2) | 33468/39 (99.9/0.1) | 0.214 |
| Liver failure (no/yes, %) | 52703/4 (100.0/0.0) | 19196/4 (100.0/0.0) | 33507/0 (100.0/0.0) | 0.034 |
| Cirrhosis (no/yes, %) | 52592/115 (99.8/0.2) | 19152/48 (99.8/0.2) | 33440/67 (99.8/0.2) | 0.277 |
| Gastritis (no/yes, %) | 52652/55 (99.9/0.1) | 19177/23 (99.9/0.1) | 33475/32 (99.9/0.1) | 0.49 |
| Diabetes (no/yes, %) | 49613/3094 (94.1/5.9) | 18360/840 (95.6/4.4) | 31253/2254 (93.3/6.7) | <0.001 |
| Dementia (no/yes, %) | 52515/192 (99.6/0.4) | 19133/67 (99.7/0.3) | 33382/125 (99.6/0.4) | 0.714 |
| Cancer (no/yes, %) | 52117/590 (98.9/1.1) | 18928/272 (98.6/1.4) | 33189/318 (99.1/0.9) | <0.001 |
| Death in hospital (no/yes, %) | 52257/450 (99.1/0.9) | 18969/231 (98.8/1.2) | 33288/219 (99.3/0.7) | <0.001 |
