# Supplementary material for: Establishment and validation of an artificial intelligence web application for predicting postoperative in-hospital mortality in patients with hip fracture: a national cohort study of 52 707 cases
Source: Int J Surg. 2024 May 15;110(8):4876–92. doi: 10.1097/JS9.0000000000001599 (PMC11325965; doi:10.1097/JS9.0000000000001599)
Supplement: Supplementary file 5 [file js9-110-4876-s006.docx]

| **Supplementary Table 3.** Subgroup analysis of clinical characteristics according to the number of comorbidities. | | | | | | |
| --- | --- | --- | --- | --- | --- | --- |
| Characteristics | Overall | Number of comorbidities | | | | p |
|  |  | 0 | 1 | 2 | ≧3 |  |
| n | 52707 | 25904 | 12299 | 11933 | 2571 |  |
| Age (%) |  |  |  |  |  | <0.001 |
| 60-69 | 13285 (25.2) | 7580 (29.3) | 3011 (24.5) | 2219 (18.6) | 475 (18.5) |  |
| 70-79 | 23093 (43.8) | 11084 (42.8) | 5399 (43.9) | 5411 (45.3) | 1199 (46.6) |  |
| 80-89 | 14520 (27.5) | 6419 (24.8) | 3479 (28.3) | 3834 (32.1) | 788 (30.6) |  |
| 90-100 | 1753 (3.3) | 792 (3.1) | 400 (3.3) | 455 (3.8) | 106 (4.1) |  |
| >100 | 56 (0.1) | 29 (0.1) | 10 (0.1) | 14 (0.1) | 3 (0.1) |  |
| Sex (male/female, %) | 19200/33507 (36.4/63.6) | 9268/16636 (35.8/64.2) | 4414/7885 (35.9/64.1) | 4433/7500 (37.1/62.9) | 1085/1486 (42.2/57.8) | <0.001 |
| Fracture type (femoral neck fracture/intertrochanteric fracture, %) | 30251/22456 (57.4/42.6) | 15242/10662 (58.8/41.2) | 7149/5150 (58.1/41.9) | 6393/5540 (53.6/46.4) | 1467/1104 (57.1/42.9) | <0.001 |
| Operation (Hip joint replacement/Internal fixation, %) | 27274/25433 (51.7/48.3) | 13152/12752 (50.8/49.2) | 6619/5680 (53.8/46.2) | 6141/5792 (51.5/48.5) | 1362/1209 (53.0/47.0) | <0.001 |
| Number of comorbidities (%) |  |  |  |  |  | <0.001 |
| 0 | 25904 (49.1) | 25904 (100.0) | 0 (0.0) | 0 (0.0) | 0 (0.0) |  |
| 1 | 12299 (23.3) | 0 (0.0) | 12299 (100.0) | 0 (0.0) | 0 (0.0) |  |
| 2 | 11933 (22.6) | 0 (0.0) | 0 (0.0) | 11933 (100.0) | 0 (0.0) |  |
| ≧3 | 2571 (4.9) | 0 (0.0) | 0 (0.0) | 0 (0.0) | 2571 (100.0) | |
| Anemia (no/yes, %) | 52362/345 (99.3/0.7) | 25904/0 (100.0/0.0) | 12229/70 (99.4/0.6) | 11738/195 (98.4/1.6) | 2491/80 (96.9/3.1) | <0.001 |
| Hypertension (no/yes, %) | 47865/4842 (90.8/9.2) | 25904/0 (100.0/0.0) | 10529/1770 (85.6/14.4) | 9528/2405 (79.8/20.2) | 1904/667 (74.1/25.9) | <0.001 |
| Coronary disease (no/yes, %) | 50865/1842 (96.5/3.5) | 25904/0 (100.0/0.0) | 11948/351 (97.1/2.9) | 10891/1042 (91.3/8.7) | 2122/449 (82.5/17.5) | <0.001 |
| Cerebrovascular disease (no/yes, %) | 50987/1720 (96.7/3.3) | 25904/0 (100.0/0.0) | 11915/384 (96.9/3.1) | 10995/938 (92.1/7.9) | 2173/398 (84.5/15.5) | <0.001 |
| Heart failure (no/yes, %) | 52541/166 (99.7/0.3) | 25904/0 (100.0/0.0) | 12284/15 (99.9/0.1) | 11843/90 (99.2/0.8) | 2510/61 (97.6/2.4) | <0.001 |
| Atherosclerosis (no/yes, %) | 52410/297 (99.4/0.6) | 25904/0 (100.0/0.0) | 12236/63 (99.5/0.5) | 11769/164 (98.6/1.4) | 2501/70 (97.3/2.7) | <0.001 |
| Renal failure (no/yes, %) | 52514/193 (99.6/0.4) | 25904/0 (100.0/0.0) | 12258/41 (99.7/0.3) | 11859/74 (99.4/0.6) | 2493/78 (97.0/3.0) | <0.001 |
| Nephrotic syndrome (no/yes, %) | 52701/6 (100.0/0.0) | 25904/0 (100.0/0.0) | 12297/2 (100.0/0.0) | 11932/1 (100.0/0.0) | 2568/3 (99.9/0.1) | <0.001 |
| Respiratory system disease (no/yes, %) | 49216/3491 (93.4/6.6) | 25904/0 (100.0/0.0) | 11395/904 (92.6/7.4) | 10093/1840 (84.6/15.4) | 1824/747 (70.9/29.1) | <0.001 |
| Gastrointestinal bleeding (no/yes, %) | 52651/56 (99.9/0.1) | 25904/0 (100.0/0.0) | 12288/11 (99.9/0.1) | 11909/24 (99.8/0.2) | 2550/21 (99.2/0.8) | <0.001 |
| Gastrointestinal ulcer (no/yes, %) | 52637/70 (99.9/0.1) | 25904/0 (100.0/0.0) | 12289/10 (99.9/0.1) | 11892/41 (99.7/0.3) | 2552/19 (99.3/0.7) | <0.001 |
| Liver failure (no/yes, %) | 52703/4 (100.0/0.0) | 25904/0 (100.0/0.0) | 12298/1 (100.0/0.0) | 11930/3 (100.0/0.0) | 2571/0 (100.0/0.0) | 0.072 |
| Cirrhosis (no/yes, %) | 52592/115 (99.8/0.2) | 25904/0 (100.0/0.0) | 12269/30 (99.8/0.2) | 11880/53 (99.6/0.4) | 2539/32 (98.8/1.2) | <0.001 |
| Gastritis (no/yes, %) | 52652/55 (99.9/0.1) | 25904/0 (100.0/0.0) | 12292/7 (99.9/0.1) | 11908/25 (99.8/0.2) | 2548/23 (99.1/0.9) | <0.001 |
| Diabetes (no/yes, %) | 49613/3094 (94.1/5.9) | 25904/0 (100.0/0.0) | 11288/1011 (91.8/8.2) | 10319/1614 (86.5/13.5) | 2102/469 (81.8/18.2) | <0.001 |
| Dementia (no/yes, %) | 52515/192 (99.6/0.4) | 25904/0 (100.0/0.0) | 12241/58 (99.5/0.5) | 11844/89 (99.3/0.7) | 2526/45 (98.2/1.8) | <0.001 |
| Cancer (no/yes, %) | 52117/590 (98.9/1.1) | 25904/0 (100.0/0.0) | 12073/226 (98.2/1.8) | 11663/270 (97.7/2.3) | 2477/94 (96.3/3.7) | <0.001 |
| Death in hospital (no/yes, %) | 52257/450 (99.1/0.9) | 25886/18 (99.9/0.1) | 12220/79 (99.4/0.6) | 11742/191 (98.4/1.6) | 2409/162 (93.7/6.3) | <0.001 |
