# Supplementary material for: Establishment and validation of an artificial intelligence web application for predicting postoperative in-hospital mortality in patients with hip fracture: a national cohort study of 52 707 cases
Source: Int J Surg. 2024 May 15;110(8):4876–92. doi: 10.1097/JS9.0000000000001599 (PMC11325965; doi:10.1097/JS9.0000000000001599)
Supplement: Supplementary file 6 [file js9-110-4876-s009.docx]

| **Supplementary Table 4.** Subgroup analysis of prediction performance for the optimal machine learning-based model. | | | | | | |
| --- | --- | --- | --- | --- | --- | --- |
| Variables | AUC (95% CI) | Threshold | Specificity | Sensitivity | Accuracy | Precision |
| Age (years) |  |  |  |  |  |  |
| 60-69 | 0.925 (0.917-0.933) | 0.305 | 0.810 | 0.893 | 0.834 | 0.661 |
| 70-79 | 0.895 (0.889-0.902) | 0.477 | 0.723 | 0.893 | 0.800 | 0.728 |
| 80-89 | 0.884 (0.876-0.892) | 0.564 | 0.745 | 0.871 | 0.824 | 0.852 |
| ≧90 | 0.928 (0.911-0.946) | 0.822 | 0.869 | 0.854 | 0.859 | 0.940 |
| Sex |  |  |  |  |  |  |
| Male | 0.898 (0.892-0.905) | 0.600 | 0.786 | 0.846 | 0.822 | 0.856 |
| Female | 0.909 (0.904-0.914) | 0.407 | 0.751 | 0.906 | 0.816 | 0.724 |
| Fracture type |  |  |  |  |  |  |
| Femoral neck fracture | 0.909 (0.904-0.914) | 0.458 | 0.735 | 0.904 | 0.819 | 0.773 |
| Intertrochanteric fracture | 0.907 (0.901-0.913) | 0.555 | 0.798 | 0.849 | 0.824 | 0.810 |
| Operation |  |  |  |  |  |  |
| Hip joint replacement | 0.898 (0.893-0.904) | 0.550 | 0.841 | 0.782 | 0.809 | 0.853 |
| Internal fixation | 0.917 (0.912-0.923) | 0.506 | 0.828 | 0.841 | 0.834 | 0.800 |
| AUC, area under curve; CI, confident interval. | | | | | | |
