# Supplementary material for: Establishment and validation of an artificial intelligence web application for predicting postoperative in-hospital mortality in patients with hip fracture: a national cohort study of 52 707 cases
Source: Int J Surg. 2024 May 15;110(8):4876–92. doi: 10.1097/JS9.0000000000001599 (PMC11325965; doi:10.1097/JS9.0000000000001599)
Supplement: Supplementary file 7 [file js9-110-4876-s013.docx]

| **Supplementary Table 5.** A comparison of evaluation performance between doctors and the AI application. | | | | | | | | | |
| --- | --- | --- | --- | --- | --- | --- | --- | --- | --- |
| Metrics | Doctors | | | | | | | | |
|  | #1 | #2 | #3 | #4 | #5 | #6 | #7 | #8 | All |
| AUC | 0.720 | 0.727 | 0.646 | 0.597 | 0.617 | 0.698 | 0.729 | 0.732 | 0.682 |
| Specificity | 0.760 | 0.800 | 0.679 | 0.630 | 0.660 | 0.736 | 0.765 | 0.814 | 0.726 |
| Sensitivity | 0.680 | 0.655 | 0.614 | 0.565 | 0.574 | 0.660 | 0.694 | 0.649 | 0.638 |
| Accuracy | 0.720 | 0.720 | 0.650 | 0.600 | 0.620 | 0.700 | 0.730 | 0.720 | 0.682 |
| Precision | 0.739 | 0.800 | 0.600 | 0.565 | 0.600 | 0.689 | 0.739 | 0.822 | 0.694 |
| AUC, area under the curve. | | | | | | | | | |
